# Avian Primordial Germ Cells Contribute to and Interact With the Extracellular Matrix During Early Migration

**DOI:** 10.3389/fcell.2019.00035

**Published:** 2019-03-28

**Authors:** David J. Huss, Sasha Saias, Sevag Hamamah, Jennifer M. Singh, Jinhui Wang, Mohit Dave, Junhyong Kim, James Eberwine, Rusty Lansford

**Affiliations:** ^1^Department of Radiology, Children's Hospital Los Angeles, Los Angeles, CA, United States; ^2^Translational Imaging Center, University of Southern California, Los Angeles, CA, United States; ^3^Department of Pharmacology, University of Pennsylvania, Philadelphia, PA, United States; ^4^Penn Genome Frontiers Institute, University of Pennsylvania, Philadelphia, PA, United States; ^5^Department of Biology, University of Pennsylvania, Philadelphia, PA, United States

**Keywords:** primordial germ cells, extracellular matrix, transcriptome, germinal crescent, quail, matrisome

## Abstract

During early avian development, primordial germ cells (PGC) are highly migratory, moving from the central area pellucida of the blastoderm to the anterior extra-embryonic germinal crescent. The PGCs soon move into the forming blood vessels by intravasation and travel in the circulatory system to the genital ridges where they participate in the organogenesis of the gonads. This complex cellular migration takes place in close association with a nascent extracellular matrix that matures in a precise spatio-temporal pattern. We first compiled a list of quail matrisome genes by bioinformatic screening of human matrisome orthologs. Next, we used single cell RNA-seq analysis (scRNAseq) to determine that PGCs express numerous ECM and ECM-associated genes in early embryos. The expression of select ECM transcripts and proteins in PGCs were verified by fluorescent *in situ* hybridization (FISH) and immunofluorescence (IF). Live imaging of transgenic quail embryos injected with fluorescent antibodies against fibronectin and laminin, showed that germinal crescent PGCs display rapid shape changes and morphological properties such as blebbing and filopodia while surrounded by, or in close contact with, an ECM fibril meshwork that is itself in constant motion. Injection of anti-β1 integrin CSAT antibodies resulted in a reduction of mature fibronectin and laminin fibril meshwork in the germinal crescent at HH4-5 but did not alter the active motility of the PGCs or their ability to populate the germinal crescent. These results suggest that integrin β1 receptors are important, but not required, for PGCs to successfully migrate during embryonic development, but instead play a vital role in ECM fibrillogenesis and assembly.

## Introduction

The extracellular matrix (ECM) plays a vital role in the timing, speed and direction of embryonic cell movements (Dufour et al., 1988; Loganathan et al., 2016). In addition to its importance in cell migration, the ECM also provides signaling cues that regulate cell behaviors and coordinate cell functions in tissue formation and homeostasis. The ECM is known to impart structure and stiffness to the developing embryo, and maintain proper tissue tension (Sato et al., 2017). The ECM helps establish and maintain stem cell niches and serves as a ligand for soluble growth factors and a system for communication between cells and tissues (Hynes, 2009; Watt and Huck, 2013; Ahmed and Ffrench-Constant, 2016; de Almeida et al., 2016). The composition of the ECM, its three-dimensional organization and proteolytic renovations are critical factors in the microenvironmental signaling that regulates cell shape, motility, growth, survival, and differentiation. At the tissue level, the ECM is known to play a dynamic role in shaping large-scale movements during early morphogenesis in Xenopus (Boucaut et al., 1990; Davidson et al., 2006), zebrafish (Latimer and Jessen, 2010), chick (Sanders, 1984), and quail (Zamir et al., 2006). The integrin receptors are a key component in a cell's ability to interact with the ECM during embryogenesis and cell migration (Beauvais-Jouneau and Thiery, 1997; Darribère et al., 2000). In addition to changing cell behavior, the binding of integrins to the ECM has a profound effect upon the ECM itself by changing the rate and timing of fibrillogenesis, assembly and breakdown (Darribère et al., 1990; Danen et al., 2002; Leiss et al., 2008).

Primordial germ cells (PGCs) represent the founder cells of the germline lineage. During amniote embryonic development the PGCs undergo a long and complex migration that moves through embryonic and extra-embryonic tissues. For example, avian PGCs are initially detected in the epiblast layer in the center of the blastoderm at the freshly laid developmental stage EGK-X (Eyal-Giladi et al., 1981). Avian PGCs then delaminate from the dorsal epiblast and move ventrally where they associate with hypoblast cells. They move with the hypoblast during gastrulation toward the anterior extra-embryonic end of the embryo to the germinal crescent (Swift, 1914). The germinal crescent is a semi-circular shaped extra-embryonic region that lies roughly along the area opaca/area pellucida (AO/AP) border anterior to the embryo (Clawson and Domm, 1969; Fujimoto et al., 1976). After ~2 days of incubation, the PGCs enter the newly formed vascular plexus and move via the circulatory system throughout the embryonic and extra-embryonic regions. PGCs leave the blood vessels around day 3 and travel along the gut mesentery to colonize the developing gonadal anlage (Nakamura et al., 2007; De Melo Bernardo et al., 2012). While there are differences between species in the routes, timing, and guidance mechanisms involved, the ability of PGCs to eventually colonize the genital ridges and produce functional sex cells is critical to reproductive success (Richardson and Lehmann, 2010; Tarbashevich and Raz, 2010; Barton et al., 2016; Cant and Laird, 2017).

Several studies have shown that migrating PGCs intimately interact with the emerging ECM as they migrate to the gonadal anlage. The migration of PGCs in relation to the spatio-temporal expression pattern of particular ECM glycoproteins has been investigated using fixed tissue histochemical and immunofluorescent approaches in mice (Soto-Suazo et al., 1999, 2002). The location and role of fibronectin along the PGC migratory route, in particular, has been examined in detail (Heasman et al., 1981; Fujimoto et al., 1985; Ffrench-Constant et al., 1991). In avians, Urven et al. (1989) described the distribution of fibronectin, laminin, chondroitin sulfate and collagen IV along the PGC migratory pathway during the first 5 days of development. PGCs may shift their adhesiveness to the different ECM substrate molecules that they encounter during their migration (García-Castro et al., 1997). Loss-of-function assays have been used to identify the roles played by individual components of the complex PGC/ECM relationship. For example, the elimination of the integrin β1 receptors in null mouse lines led to poor colonization of the gonad by migratory PGCs (Anderson et al., 1999). Knockout mouse lines lacking the Msx1/2 transcription factors showed abnormal PGC migration along with increased amounts of fibronectin (Sun et al., 2016). These previous studies have led to an increasing understanding of PGC-ECM interactions particularly during the later migratory period; however, the early migratory stages have received less study.

Direct visualization of PGC/ECM interactions in real time *in vivo* may provide new insights into the early migratory phase of PGCs. Unfortunately, imaging PGC migration in early mouse embryos is technically challenging (Molyneaux et al., 2001). We have previously demonstrated the utility of fluorescently labeled transgenic quail for imaging early developmental processes (Sato et al., 2010; Huss et al., 2015a; Bénazéraf et al., 2017). Recently, we have developed a novel transgenic quail line that is a useful model system for studying early cell motility and migration. A shared characteristic of all of the ubiquitously expressing transgenic quail lines is that slowly dividing cells, such as PGCs, show a higher level of fluorescence compared to rapidly dividing cells. This unique feature allows us to follow the movement of PGCs *in vivo* (Huss et al., 2015a). The injection of fluorescently labeled anti-glycoprotein antibodies into live embryos makes following the highly dynamic ECM possible (Little and Drake, 2000; Filla et al., 2004). By combining these approaches, we can now visualize the interactions of PGCs with their surrounding ECM in real time.

Interpreting these PGC-ECM interactions will require a thorough understanding of the molecular profile of these unique stem cells. The previously published PGC mRNA abundance data have primarily been obtained from stem cell culture (Macdonald et al., 2010; Jean et al., 2015). Knowing which ECM genes are expressed in avian PGCs and their surrounding somatic cells at different stages along their migratory pathway will be vital in understanding *in vivo* PGC/ECM interactions.

Numerous “matrisomes” have recently been compiled that list all of the genes that code for the structural ECM components in a particular species. Using domain-based organization, genes that code for proteins that directly interact with or remodel the ECM have been included (Naba et al., 2016). These lists also include genes that have the potential to interact with the structural ECM (Naba et al., 2012). To account for these broad categories, Hynes and Naba (2012) defined the Core Matrisome to include all of the ECM glycoproteins, collagens, proteoglycans and a second category as matrisome associated genes that includes ECM-affiliated proteins, ECM regulators, and secreted factors. Extensive transcriptome and proteome data have been included within the searchable human and mouse matrisomes. To date, *D. rerio, C. elegans*, and *D. melanogaster* matrisomes have also been compiled, but avian matrisomes are lacking (http://matrisome.org/).

Here, we fill the gaps in knowledge concerning certain aspects of PGC-ECM interactions by using several methodologies. First, we compiled a quail matrisome and used it to analyze the transcriptome from individual PGCs manually isolated from early embryonic stage transgenic quail. The single cell RNA-seq (scRNA-seq) data shows that PGCs express a complex mixture of ECM and ECM-affiliated genes including fibronectin and numerous members of the collagen, laminin, and integrin gene families. To confirm the scRNA-seq results, whole mount mRNA fluorescent *in situ* hybridization (FISH) and immunofluorescence (IF) of selected genes were combined with confocal imaging to localize these transcripts and proteins to the PGCs and the surrounding cells of the germinal crescent. Live imaging of transgenic quail injected with anti-fibronectin or laminin antibodies showed rapid cell shape changes of PGCs in the germinal crescent and their active interplay with a dynamic ECM. Perturbation of the β1 integrin receptor/ECM interaction had no discernable effect on PGC cell motility, but resulted in a dramatic reduction in the abundance of the matrix fibril meshwork in the germinal crescent.

## Materials and Methods

### Transgenic Quail

Three separate transgenic Japanese quail (*Coturnix japonica*) lines were utilized in this study. We previously reported the [Tg(PGK1:H2B-cherryFP)] or [PGK1] line that fluorescently labels all cell nuclei and allows cell proliferation rates to be determined (Huss et al., 2015a). The [Tg(UbC.H2B-cerFP-2A-Dendra2)] or [UbC.CerD2] quail line ubiquitously co-expresses histone 2B-ceruleanFP (H2B-cerFP) and Dendra2. Dendra2 is a photoconvertible green fluorophore that efficiently converts to red after exposure to near-UV light (Gurskaya et al., 2006). Details on the molecular cloning, lentivirus production and EGK-X blastoderm injections have been reported (Huss and Lansford, 2017). The [Tg(hUbC:Membrane-eGFP)] quail line was a kind gift from Dr. Jerome Gros (Pasteur Institute, Paris, France) (Saadaoui et al., 2018) and labels the plasma membrane of all cells. All animal procedures were carried out in accordance with approved guidelines from the Children's Hospital Los Angeles and the University of Southern California Institutional Animal Care and Use Committees.

### Japanese Quail Genome

For these studies we used the *Coturnix japonica* genome (https://www.ncbi.nlm.nih.gov/genome/?term=coturnix).

### Quail Matrisome

We screened the human matrisome genes against the *Coturnix japonica* genome to identify gene orthologs that could be considered as the Quail matrisome. The matching quail genes were divided into (1) “Core matrisome” and (2) “Matrisome-associated” genes similar to the human matrisome (Naba et al., 2016). Categories within groups—(1) Core matrisome: ECM, Collagens, and Proteoglycans; (2) Matrisome-associated: ECM-related, Regulators, and Secreted Factors.

### Transcriptome Analysis of Individual PGCs

[Tg(PGK1:H2B-mCherry)] quail embryos were incubated until HH3, HH6, or HH12 and harvested on paper rings into PBS. Tissue areas containing PGCs were excised using fine iris scissors (Figure S1). The tissues were dissociated at 37°C using TrypLE Express (ThermoFisher), pipet triturated and diluted in PBS. A drop of diluted cell suspension was placed on a plastic petri dish on the stage of an Olympus MVX10 epi-fluorescent stereomicroscope. Using a micro manipulator and alternating between bottom white light illumination and CY3 filtered fluorescence, single cells were visually aspirated into glass needles with a tip opening of ~40 μm. Once aspirated, the glass pipet was quickly withdrawn from the suspension. The tip of the glass pipet containing the single cell was crushed into the bottom of a 1.5 ml RNase/DNase free microfuge tube. Twenty cells from each developmental stage were collected based on their H2B-mCherry brightness and overall size. Amplification of the mRNA population was then carried out according to the protocol outlined in Morris et al. (2011). Deep sequencing of the prepared single cell libraries was performed at the University of Pennsylvania Genomics Center. Bioinformatics processing was carried out in the laboratory of Dr. Junhyong Kim using their software platform Interactive Data Visualization [KimLabIDV, v1.03, now PIVOT] (Zhu et al., 2018). RNAseq reads were aligned to the reference *Coturnix* and *Gallus* genomes using Bowtie2 (Langmead and Salzberg, 2012) or the Star software package (Dobin et al., 2013). Outlier cells were excluded based on cell library read depth (<50 million reads/cell) and overall percentage sequence alignment. Unsupervised clustering analysis of the scRNAseq data was used to define cell types and stable states (Kim and Eberwine, 2010). Cells were categorized using principle component analysis and assigned unique molecular identifiers (UMI). DDX4 and DAZL were used as PGC marker genes to screen the 20 picked cells from each developmental stage. We identified 12 PGCs from HH3 (60%), 13 PGCs from HH6 (65%), and 8 PGCs from HH12 (40%). Quail gene orthologs to the mouse and human core matrisome genes (Naba et al., 2016) were identified and used to assign scRNAseq reads to particular genes. Gene sets were analyzed and graphed using Microsoft Excel.

### Immunofluorescence and Static Imaging

Anti-fibronectin (B3/D6), anti-laminin (31-2), anti-fibronectin/laminin receptor (JG-22), anti-integrin β1 subunit (CSAT), anti-fibrillin 2 (JB-3), anti-collagen type IV (M3F7) and anti-chondroitin sulfate (9BA12) monoclonal antibodies were purchased from the Developmental Studies Hybridoma Bank at the University of Iowa (DSHB, Iowa City, IA). Directly conjugated B3/D6 (AlexaFluor 594) and 31-2 (AlexaFluor 555) were kind gifts from Dr. Charles Little (University of Kansas Medical School, Kansas City, MO). Anti-CVH polyclonal antibody (also known as VASA or DDX4) was a kind gift of Dr. Toshiaki Noce, Keio University School of Medicine, Tokyo, Japan). The anti-DAZL rabbit monoclonal antibody (ab215718) was purchased from Abcam (Cambridge, MA). Before use, the CSAT antibody serum was concentrated from 20 to 174 μg/ml by centrifugation through a 30,000 mw cutoff Amicon Ultra-15 cellulose filter column (Millipore) at 4,000 × g for 1 h at 4°C.

Eggs were grown in a forced air humidified incubator at 37°C until the proper age. Quail embryos were staged according to Eyal-Giladi and Kochav (1976), Hamburger and Hamilton (1951) and Ainsworth et al. (2010) with additional detailed descriptions of primitive streak morphology and staging from Streit and Stern (2008). Embryos were harvested from the yolk using a filter paper ring (Chapman et al., 2001), washed in PBS (phosphate buffered saline) and fixed in 4% formaldehyde/PBS made from a 36% stock solution (Sigma, F8775). Embryos were dissected free of the paper ring, washed in PBST (0.1% Triton X-100), blocked in PBST containing 1% bovine serum albumin and 5% normal donkey serum and incubated in primary antibodies in blocking solution overnight at 4°C. DSHB antibodies were diluted to 5 μg/ml. Anti-CVH and DAZL were diluted to 1:500. After washing, the embryos were blocked in PBSTW (0.1% Tween-20) and incubated in 1:1,000 dilutions of appropriate donkey secondary antibodies conjugated to AlexaFluor fluorophores (Thermo-Fisher) overnight at 4°C. After washing in PBST, embryos were incubated in PBST + 0.05 μg/ml DAPI for 1 h at 4°C, washed again and cleared in ScaLe U2 solution (Hama et al., 2011). Controls in which the primary antibody was eliminated failed to show specific labeling. Embryos were stored in the dark at 4°C until imaging. Static imaging was conducted with the cleared embryos placed dorsal side down on No. 0 glass bottom petri dishes (Mattek, Ashland, MA) and covered with a thin layer of ScaLe U2. Imaging was performed on a Zeiss 780 inverted confocal microscope with 5x/0.16NA, 10x/0.45NA, 20x/0.8NA, 63x/1.4NA oil Plan-Apochromat objectives or a 40x/1.1NA water LD C-Apochromat water immersion objective. Images were processed using the Zeiss Zen software along with NIH Image J (PMID 22743772). Slight adjustments to the contrast and brightness of the entire image were occasionally made.

### Hybridization Chain Reaction

*In situ* hybridization was carried out on whole-mount quail embryos using the hybridization chain reaction (HCR) technique (Choi et al., 2014, 2016; Huss et al., 2015b). Oligonucleotide antisense probes (30 or 50 nt) against quail or chicken target sequences were commercially synthesized (IDT or ThermoFisher, see Video S4 for the exact sequences). Probes sets contained between 4 and 11 probes and included unique initiator sequences to allow for simultaneous multiplex hybridizations. Probes were hybridized to the embryonic mRNA target sequences overnight at 37°C. After washing, fluorescently conjugated hairpin amplifiers were hybridized to the probes overnight at RT. Controls included the elimination of both probes and hairpins to test the inherent fluorescence of the embryonic tissue and the use of only hairpins to judge whether they were contributing to non-specific signal production. All HCR-treated embryos were imaged similar to the aforementioned IF-treated embryos.

### Time-Lapse Imaging and Microinjection

Fertilized Tg[hUbC:H2B-Cerulean-2A-Dendra2] quail eggs were incubated on their sides until HH3 then windowed. Injections of directly conjugated anti-fibronectin (abB3/D6) or anti-laminin (ab31-2) antibodies [1 mg/ml] were made into 3 locations around the anterior area opaca/area pellucida boundary (Little and Drake, 2000). The window was then sealed with parafilm and the egg incubated 2 h until being harvested on a paper ring. Embryos were cultured dorsal side down on No. 0 glass bottom 35 mm petri dishes that had been covered by a thin layer of agar/albumin (Sato and Lansford, 2013). A ring of moist Kimwipe (Kimtech Science) was wrapped around the inside perimeter of the dish and the top sealed with a thin strip of parafilm. The cultured embryo was allowed to settle for 1 h in the microscope stage mounted incubator at 37°C before imaging. Confocal Z stacks were taken at 1.5 min time intervals in order to capture short-term cell morphology changes. In the CSAT live imaging experiments, the embryos were first imaged as above then were removed from the microscope incubator, injected with CSAT [174 μg/ml] antibodies just under the epiblast, lateral to the primitive streak at the level of Hensen's node and returned to the heated stage for 1–2 h before additional imaging.

## Results

### Quail Matrisome

**Figure 1:**
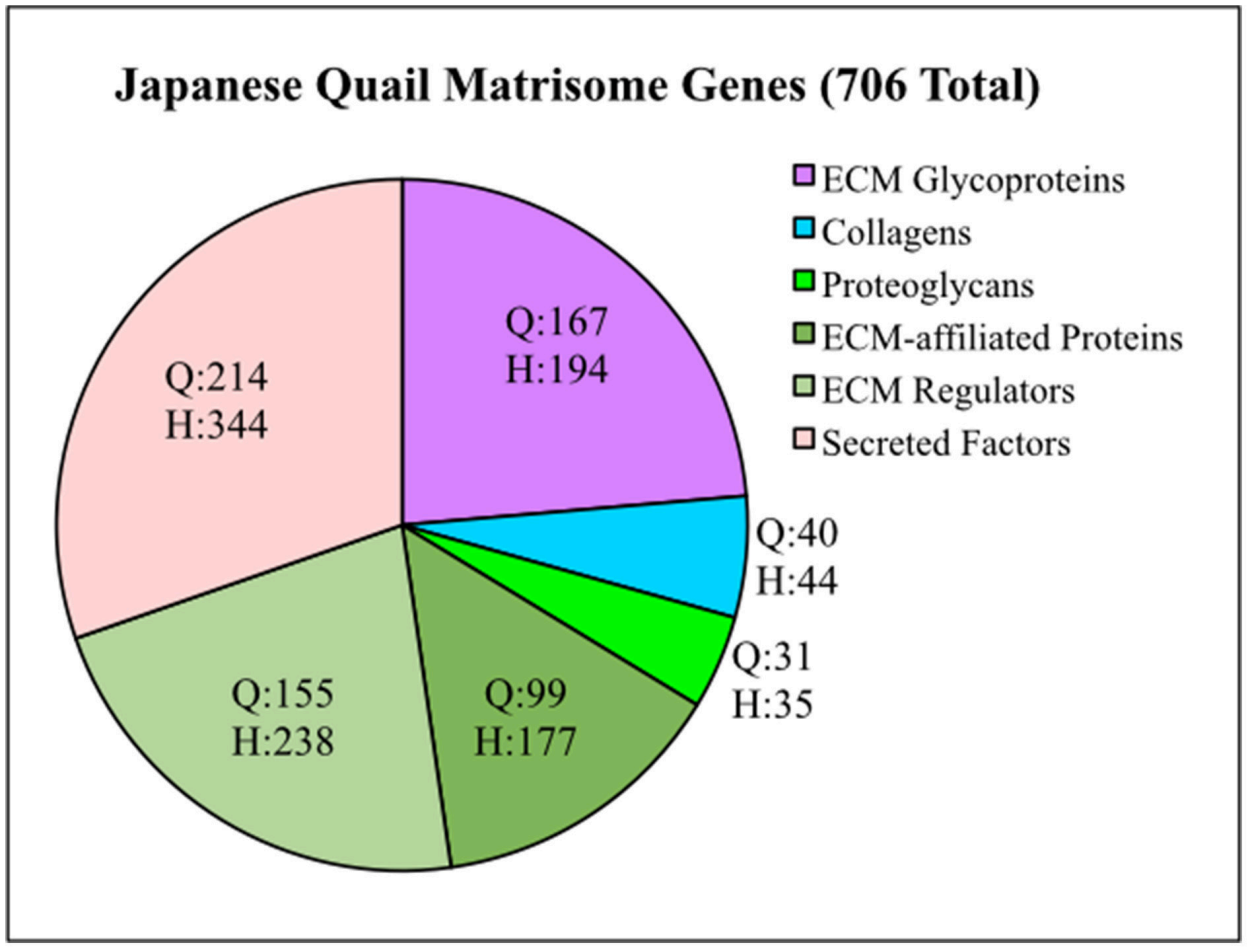
Quail matrisome categorized according to the human matrisome results of Naba et al. (2016). Q = Quail genes (706 total). H = Human genes (1,026 total). ECM Glycoproteins, Collagens and Proteoglycans make up the Core Matrisome. ECM-affiliated Proteins, ECM Regulators and Secreted Factors are Matrisome Associated genes.

To generate a Quail Matrisome (Table S1), we used a bioinformatic approach to screen the *Coturnix* genome for ECM and ECM-associated genes modeled after the human matrisome (Naba et al., 2016). Approximately 706 quail matrisome genes were identified compared to 1,026 in the human (Figure 1). The quail Core matrisome category contains 167 ECM glycoprotein, 31 proteoglycan, and 40 collagen genes. The quail Matrisome-associated category consists of 99 ECM-affiliated proteins, 155 ECM regulators and 214 secreted factors. Full bioinformatics information for the quail matrisome genes can be found in Table S1.

### ECM and ECM-Associated Transcripts Are Expressed by PGCs

Since avian PGCs undergo a long and complex migratory pathway across the first three days of post-laying development, we hoped to understand how their ECM transcriptome changed during this time. The inherent brightness of PGC nuclei in the Tg(PGK1:H2B-mCherry) quail line along with their unique morphology (large diameter, globular shape with internal glycogen vesicles) allowed us to pick single cells from enzymatically dissociated tissue. PGCs from HH3, HH6, and HH12 embryos were collected at distinct morphological regions of the embryos that included the germinal crescent (Figure S1). Amplification of the mRNA was performed according to the protocol described by Morris et al. (2011). Next generation sequencing produced a transcriptome that was analyzed based on the newly generated quail matrisome. The scRNAseq raw data for the quail matrisome genes are provided in Table S2.

**Figure 2:**
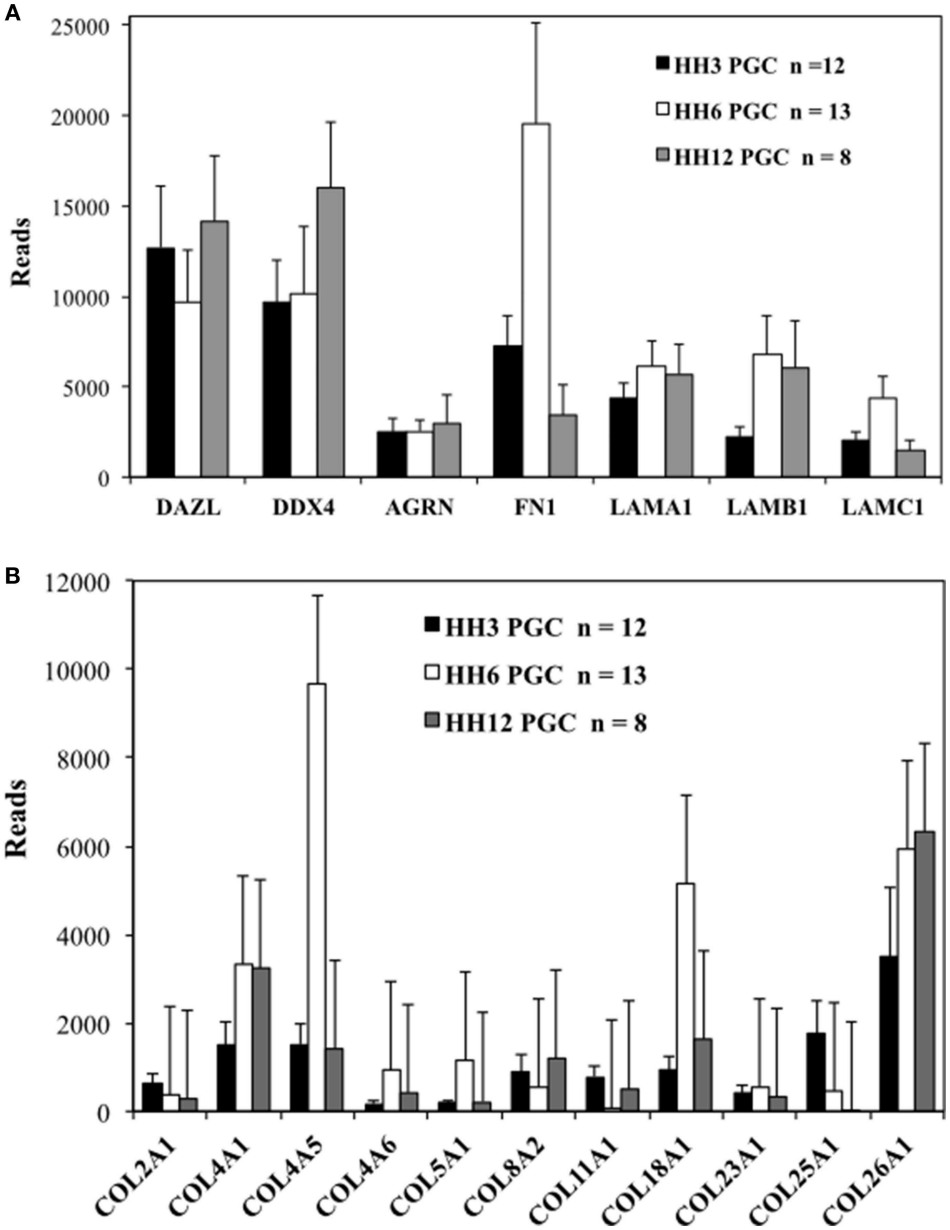
Quail PGCs express mRNA for a diverse set of matrix glycoprotein and collagen genes. scRNAseq of select ECM transcripts in PGCs at HH3, HH6, and HH12. **(A)** Matrix genes along with PGC markers DAZL and DDX4. DAZL = Deleted in azoospermia-like. DDX4 = (Dead-box helices 4 or chicken vasa homolog (CVH). AGRN, Agrin; FN, Fibronectin; LAMA1, Laminin alpha subunit 1; LAMB1, Laminin beta subunit 1; LAMBC1, Laminin gamma subunit 1. **(B)** Collagen genes. Y axis scaling has been adjusted separately in **(A,B)**. Bars represent the mean + s.e.m. The number of single cells in each group are shown in the legend.

**Figure 3:**
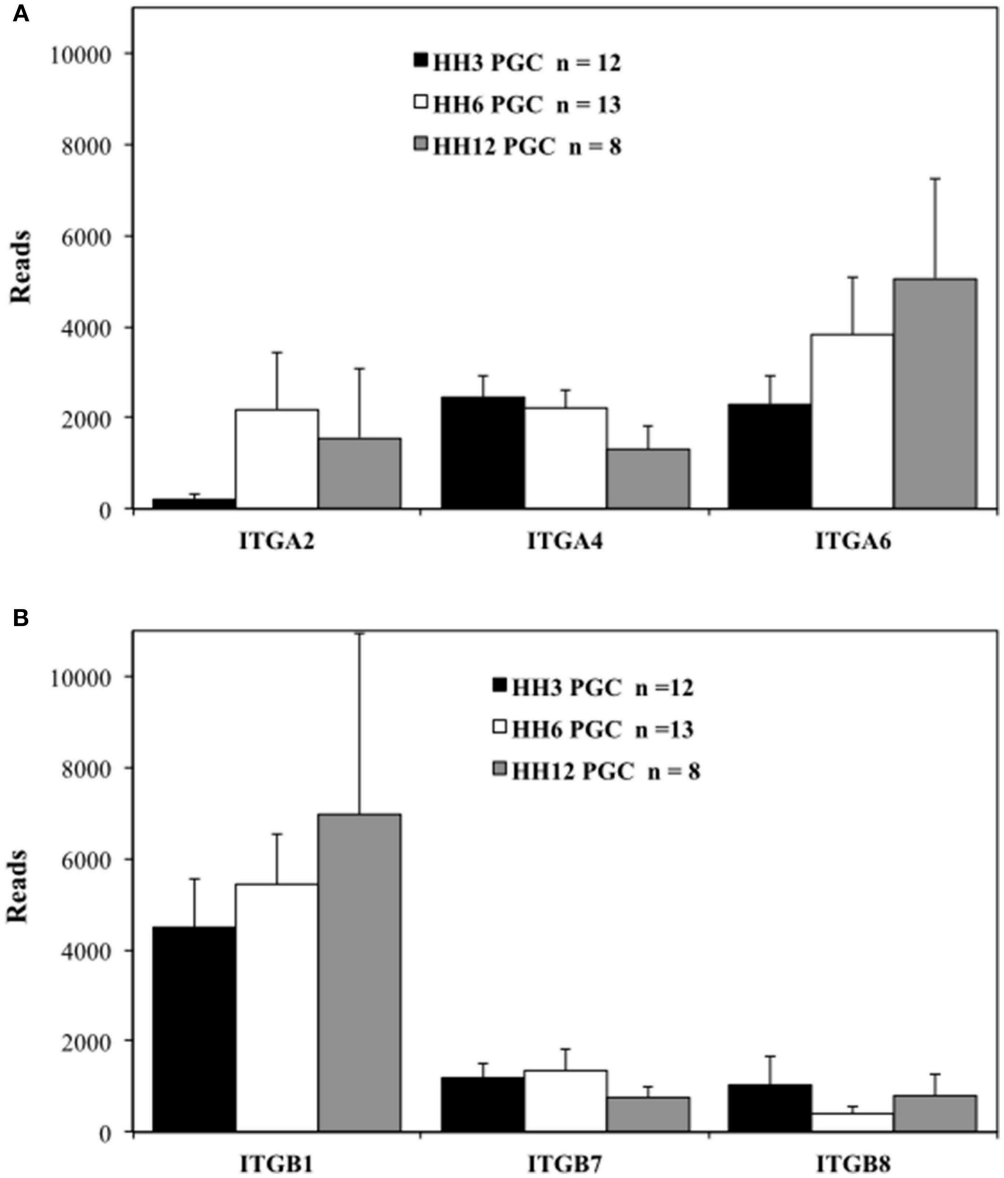
Migratory quail PGCs express multiple integrin alpha and beta subunits. scRNAseq of integrin receptor subunit mRNA transcripts from PGCs across 3 developmental time points. **(A)** Integrin alpha subunits (ITGA2, 4, and 6). **(B)** Integrin beta subunits (ITGB1, 7, and 8). Bars represent the mean + s.e.m. The number of single cells in each group are shown in the legend.

While not strictly quantitative, the number of sequencing reads, likely provides a rough indication of overall mRNA abundance. The genes DAZL (deleted in azoospermia like) and DDX4 (DEAD-box helicase 4, also referred to as chicken vasa homolog or CVH) were used as markers for PGCs. The number of reads for matrix glycoproteins, collagens and integrin receptor subunits was compared for PGCs across three developmental time points (Table S2 and Figures 2, 3). PGCs express many of the core matrisome glycoproteins with fibronectin 1 (FN1), laminin α1 subunit (LAMA1), laminin β1 subunit (LAMB1), and laminin γ1 subunit (LAMC1) showing the highest average number of reads per PGC. With the exception of FN1 at HH6, the abundance of these glycoprotein transcripts does not change markedly across developmental time points (Figure 2A). Additional matrix mRNA transcripts (shown in Figure S5D) such as the fibulin (FBN) and fibrillin (FBLN) families and heparan sulfate glycoprotein (HSPG) are clearly present but appear at much reduced levels compared to fibronectin and laminin. Many types of collagen mRNA transcripts were also detected in PGCs (Figure 2B). COL4A1, COL4A5, and COL26A1 were elevated slightly in expression while COL18A1 showed an increased number of reads at HH6 (Figure 2B).

The integrin ITGA2, ITGA4, and ITGA6 alpha subunits are detected at high levels in PGCs across the three time points examined (Figure 3A). ITGA2 shows a distinct increase in abundance between HH3 and HH6. Among the integrin β subunits, ITGB1subunit transcripts appear most numerous, with the ITGB7 and ITGB8 subunit showing lower, but consistent expression at the three developmental stages examined (Figure 3B). The integrin receptor subunits that showed lower relative levels of expression are shown in Figure S5. ITGA1 and ITGAV subunits were present, but at much reduced levels compared to ITGA2, ITGA4, and ITGA6 (Figure S5A). ITGB2, ITGB3, ITGB4, ITGB5, and ITGB6 subunit transcripts were amplified at the highest relative numbers at HH6 although, overall, these subunits were at lower levels than ITGB1 (Figure S5B). Note the change in the y-axis scale between Figure 3B and Figure S5B.

While integrin receptors, especially those containing the ITGB1 subunit, are highly abundant at the mRNA transcript level in PGCs, non-integrin receptors may also play important roles in cell/ECM interactions. Transcript levels for non-integrin receptors are shown in Figure S5C. Syndecan (SDC2), hyaluronan mediated motility receptor (HMMR) and CD47 were present in levels comparable to the most common integrin receptors. Dystroglycan (DAG1) was particularly abundant in PGCs at HH6 (Figure S5C).

### PGCs Associate Closely With Fibronectin and Laminin

**Figure 4:**
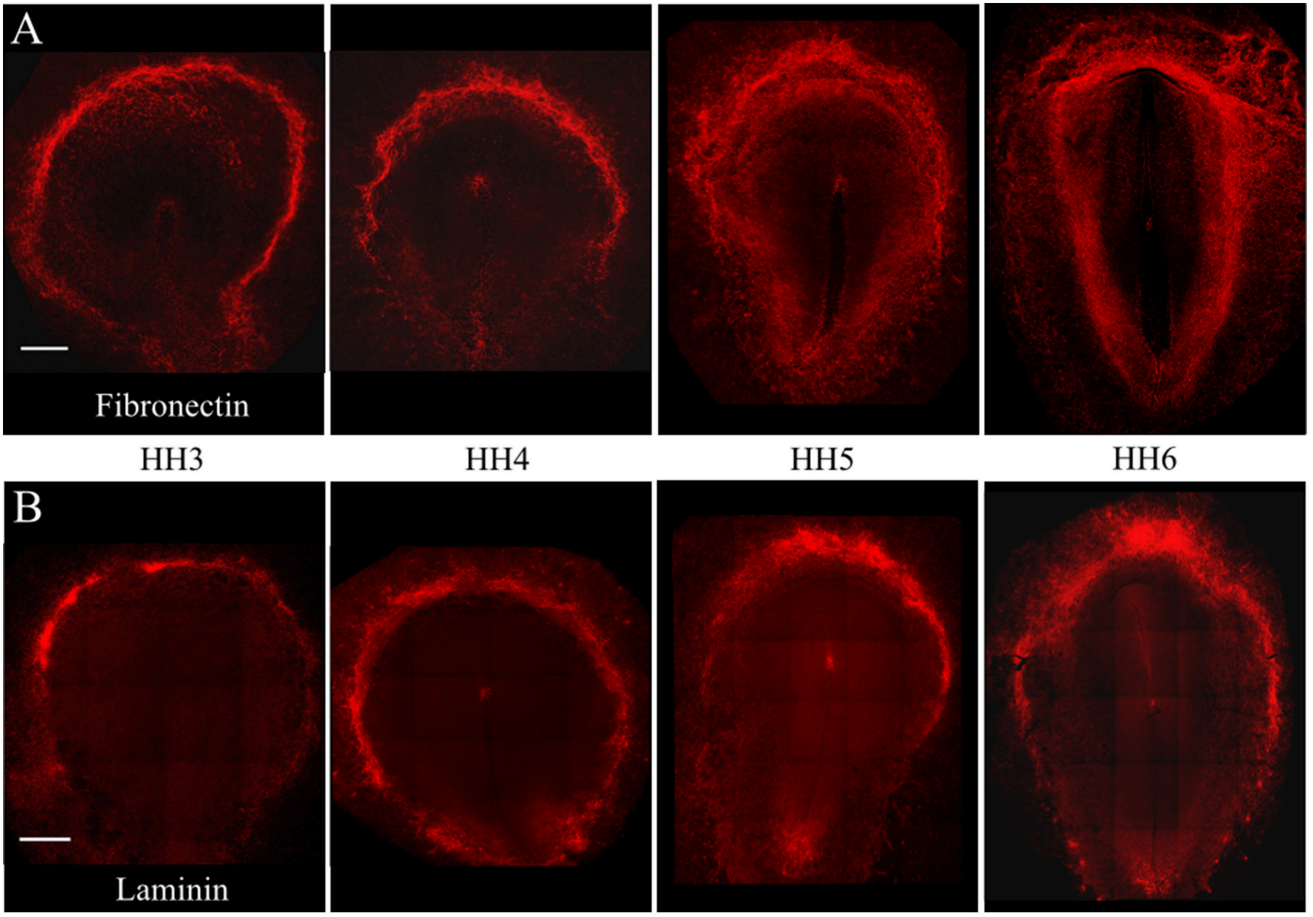
Fibronectin and Laminin protein is highly expressed at the AO/AP border and germinal crescent at stages HH3-6. Wholemount immunofluorescence for fibronectin (abB3/D6) and laminin (ab31-2) in 4 developmental stages. **(A)** Fibronectin (red), **(B)** Laminin (red). Images are maximum intensity projections of 10x tiled confocal Z stacks taken from the dorsal aspect. Anterior (rostral) to top. Scale bars = 500 um.

**Figure 5:**
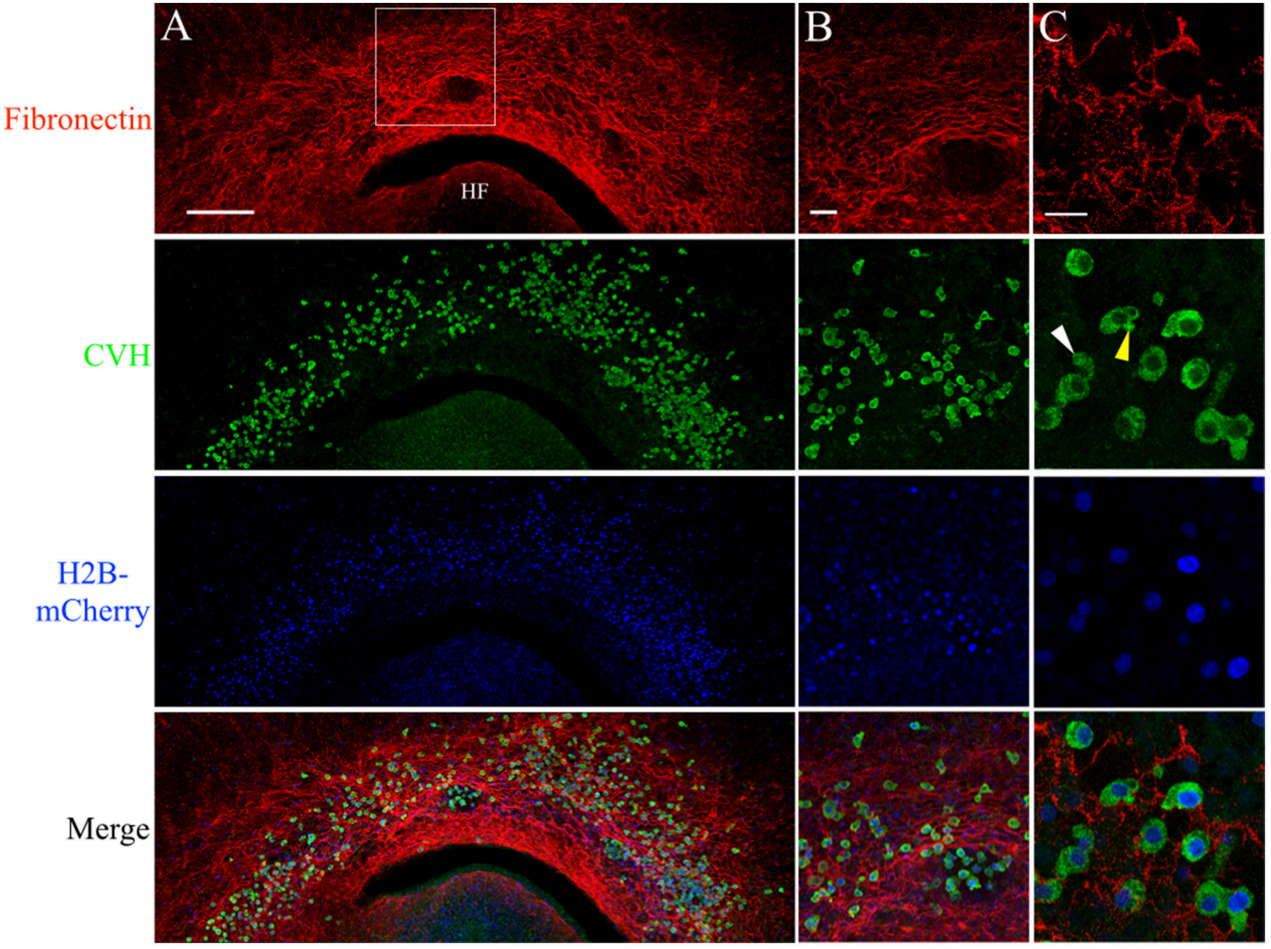
PGCs are closely associated with the complex fibronectin fibril meshwork in the germinal crescent. Wholemount immunofluorescence for fibronectin (abB3/D6) and CVH across the germinal crescent of HH5 and HH6 [Tg(PGK1:H2B-mCherry)] quail embryos. **(A)** Fibronectin red, CVH (PGC marker, green), H2B-mCherry (blue) and merged maximum intensity projections of 10x tiled confocal Z stacks taken from the dorsal aspect of an HH6 embryo germinal crescent. Anterior (rostral) to top. HF = head fold. **(A)** Scale bar = 250 μm. Images in **(B)** are maximum intensity projections of 20x tiled confocal Z stacks taken from the dorsal aspect at the location of the bounding box in **(A)**. **(B)** Scale bar = 50 μm. **(C)** Images are maximum intensity projections of 4 × 2.1 μm optical sections from 63x confocal Z stacks taken in an HH5 embryo. Yellow arrow in the CVH image highlights a bleb on a PGC. White arrow indicates a long cellular process. Scale bar = 25 μm.

Individual PGCs strongly express FN1, LAMA1, LAMB1, and LAMC1 transcripts at developmental stages HH3, HH6, and HH12. To investigate the intimate associations between nascent, migrating PGCs and these ECM components, we used immunofluorescence (IF) to detect extracellular fibronectin using the B3/D6 antibody on whole-mount quail embryos ranging from stage HH3 to HH6 (Figure 4A). Confocal 3D (x, y, z) imaging across the entire embryo revealed that fibronectin protein expression was most highly concentrated at the border of the area opaca (AO) and area pellucida (AP) in all four stages shown. Expression at the anterior (rostral) end of the AO/AP border in the germinal crescent where PGCs congregate contained a dense meshwork of FN fibrils (Figures 4A, 5A). In agreement with earlier studies, the expression pattern of FN in the embryonic tissue layers was less dense than at the AO/AP border, consisting of short, scattered fibrils and puncta (Duband and Thiery, 1982; Raddatz et al., 1991).

Anti-FN IF revealed an intricate mesh of FN fibrils in the HH6 germinal crescent (Figure 5A). IF using an antibody against the cytoplasmic PGC marker chick vasa homolog (CVH) shows that a large number of PGCs had migrated into the same region. In addition to IF, PGCs were also localized by the bright cell nuclei of [PGK1] transgenic embryos. Within the germinal crescent of some embryos, bulges, or folds, of the tissue layers created open spaces devoid of most cells (Sanders, 1982). An area such as this is shown in Figure 5B. Interestingly, PGCs were often found in these spaces while neither fibronectin nor laminin was present. Antibodies against fibrillin-2 (JB-3), collagen type IV (M3F7) and chondroitin sulfate (9BA12) also failed to label these spaces (data not shown), yet PGCs can be seen migrating throughout these germinal crescent spaces. The ECM component within these spaces remains to be determined. CVH IF showed a high proportion of PGCs, whether inside of these spaces or not, as having morphological characteristics consistent with active cell motility such as blebs (yellow arrow) and long cellular processes (white arrow, Figure 5C. See also Video S1). At stage HH12, PGCs in the germinal crescent continue to be in close contact with the FN fibril meshwork (Figures S2).

**Figure 6:**
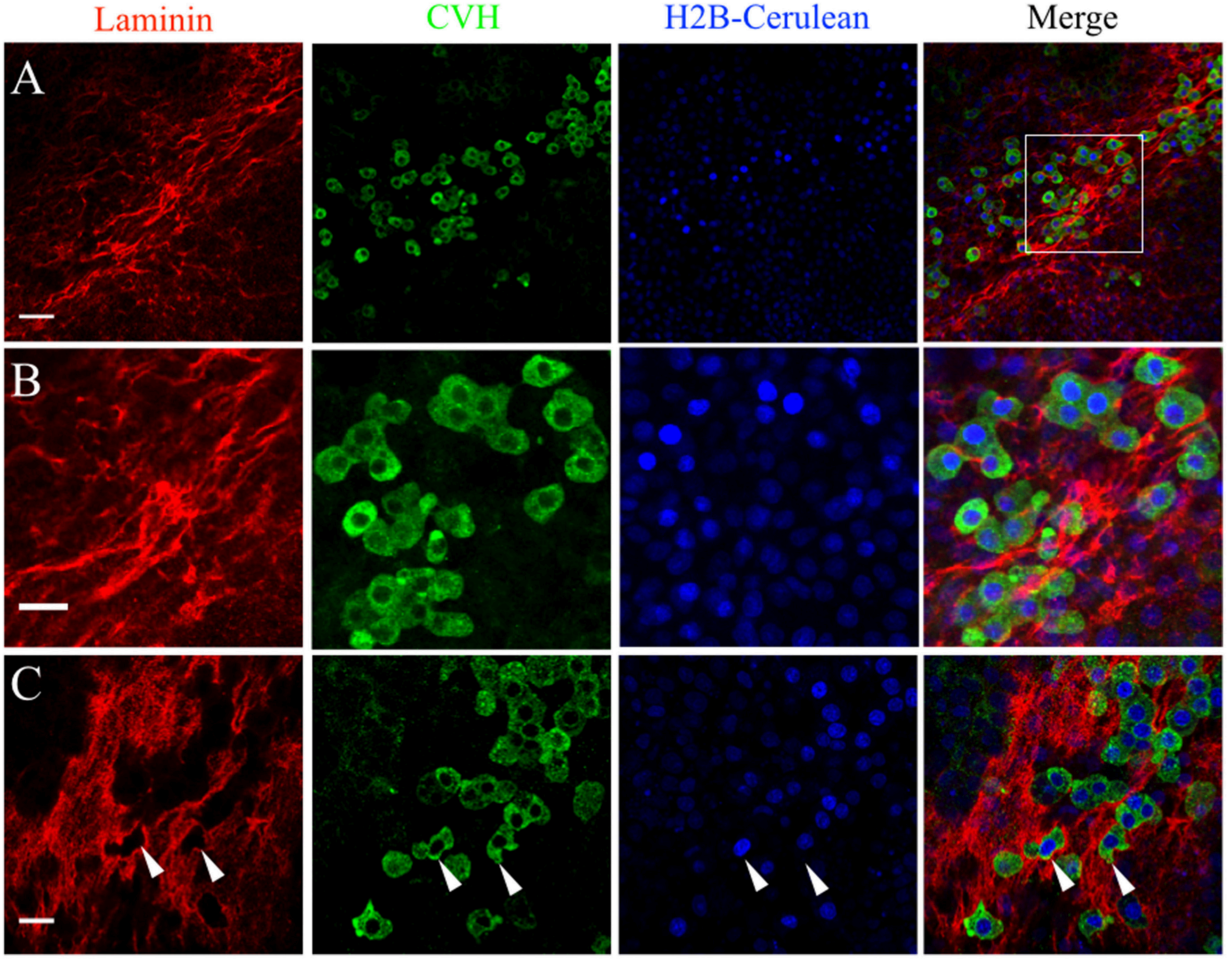
PGCs in the germinal crescent are intimately associated with the laminin fibril meshwork. Whole-mount immunofluorescence for laminin (ab31-2) and CVH from two locations in the lateral germinal crescent of an HH5 [Tg(hUbC:H2B-Cerulean-2A-Dendra2)] quail embryo. Images in **(A)** are single optical sections (3.2 μm Z depth) taken at a lateral region of the germinal crescent using a 20x 0.8NA objective. Scale bar = 50 um. The bounding box in the merge indicates the region shown in **(B)** at increased magnification. Scale bar = 25 μm. **(C)** Images are maximum intensity projections of 3 optical sections (2.3 μm each) from 40x confocal Z stacks taken from the dorsal aspect of the embryo at the lateral germinal crescent. White arrows in **(C)** highlight regions of laminin that appear completely displaced by a PGC. Scale bar = 25 μm.

The pattern of laminin glycoprotein expression was assayed in HH3-HH6 whole-mount quail embryos using the 31-2 antibody (Figure 4B). Like FN, laminin expression was most abundant at the AO/AP border and within the germinal crescent (Figure 4B). Laminin was also detected in the basal lamina, or basement membrane, on the ventral surface of the epiblast layer lateral to the primitive streak at HH5, which agrees with previous reports (Bortier et al., 1989; Zagris et al., 2000). The majority of PGCs in the germinal crescent closely associated with the areas highest in laminin fibril density (Figure 6, Merge). Much like fibronectin, laminin fibrils formed a tight meshwork in the HH5 germinal crescent and surrounded or enclosed, some PGCs (Figure 6C white arrows). PGCs were seen clustered together in clumps or individually, many with smooth, globular morphology.

### Validation of PGC Transcriptome Results With IF and FISH in Embryos

**Figure 7:**
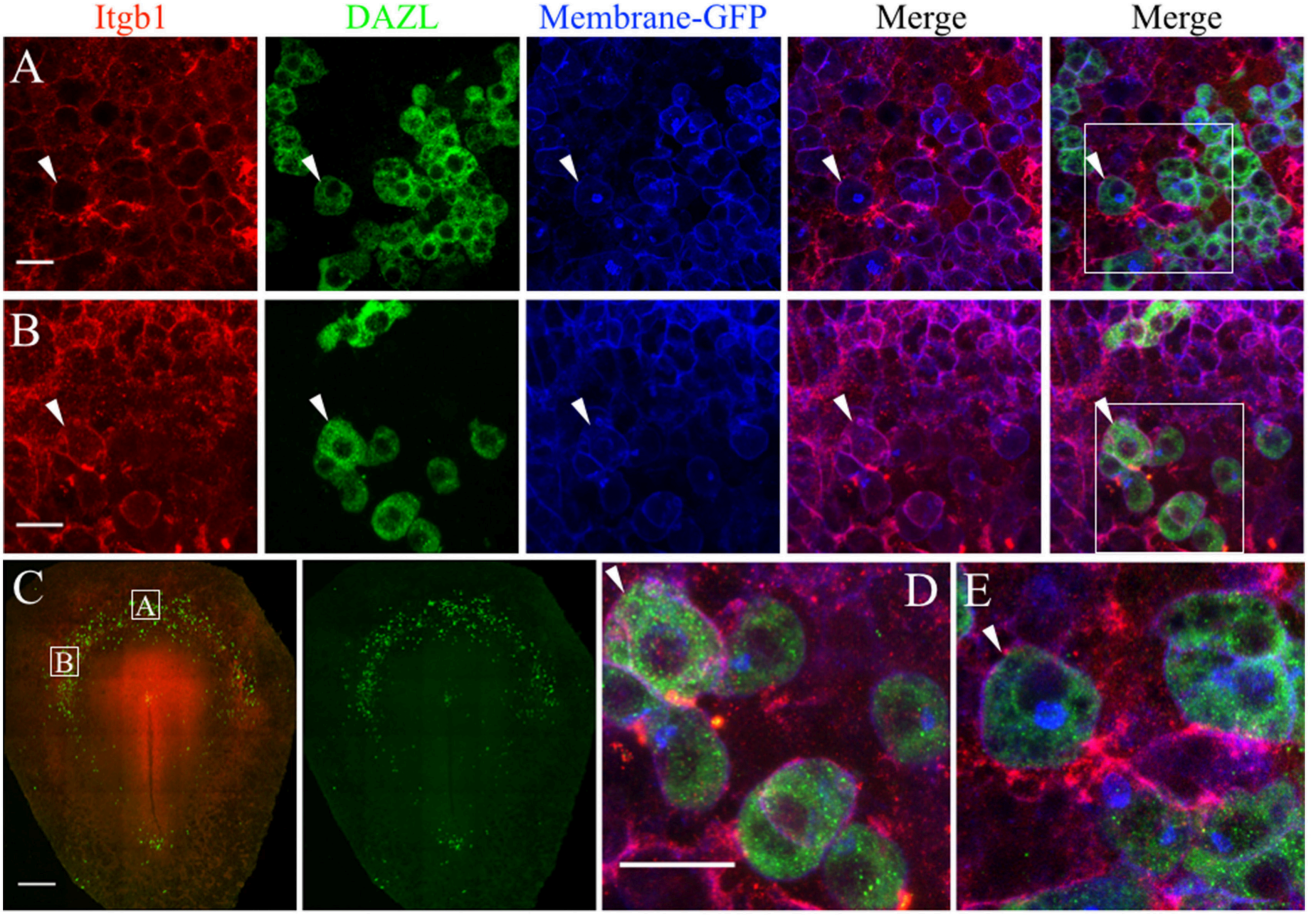
PGCs and their surrounding cells in the germinal crescent express integrin beta 1 subunit protein on their cell surfaces. Whole-mount immunofluorescence for fibronectin and laminin receptors (integrin beta 1 subunit, abJG-22) and PGC-specific Dazl at two separate locations within an HH4 [Tg(hUbC:Membrane-eGFP)] quail embryo germinal crescent. **(A)** Central germinal crescent (see panel C for location). Left to right: Integrin beta 1 subunit (Itgb1, abJG-22, red), Dazl (PGC marker, green), membrane-GFP (blue), Itgb1 and membrane-GFP merge, Itgb1, membrane-GFP, Dazl merge (bounding box shown at higher magnification in E). White arrows indicate one PGC imaged in cross section with a clearly defined perimeter of Itgb1. 20x 0.8x zoom. **(B)** Lateral germinal crescent (see panel C for location). Left to right: Integrin beta 1 subunit (Itgb1, abJG-22, red), Dazl (PGC marker, green), membrane-GFP (blue), Itgb1 and membrane-GFP merge, Itgb1, membrane-GFP, DAZL merge (bounding box shown at higher magnification in D). White arrows indicate one PGC with a clearly defined perimeter of Itgb1 that was imaged at an oblique angle. 20x 1.0x zoom. **(C)** Tiled 10x confocal Z stack maximum intensity projections of the whole-mount HH4 embryo. Bounding boxes indicate location of images in **(A,B)**. Itgb1 (red), Dazl (green). Scale bar C = 500μm. **(D,E)** Images are digital zooms of bounding boxes in **(B,A)** Merge. White arrows in **(D,E)** highlight particular PGCs with integrin beta 1 subunit receptor labeled puncta on the cell surface which was imaged in oblique **(D)** or in cross-section **(E)** by the confocal optical slice. Scale bar A, B, D, E = 25 μm.

We used whole-mount immunofluorescence and *in situ* hybridization to validate the scRNAseq expression data of select ECM genes in migratory stage PGCs. JG-22 is an antibody specific for the integrin β1 subunit that makes up part of the heterodimeric cell surface receptors that bind laminin and fibronectin (Greve and Gottlieb, 1982). This antibody showed ubiquitous expression across all embryonic and extra-embryonic tissues in primitive streak stage embryos (Figure 7C). Dazl+ PGCs in HH4 [Tg(hUbC:Membrane-eGFP)] quail embryos showed strong co-localization of integrin β1 with their GFP+ cell membranes (white arrows, Figures 7A,B,D,E). Somatic cells in close proximity to the PGCs in the germinal crescent were also strongly positive for integrin β1 subunit protein (Figures 7A,B,D,E).

**Figure 8:**
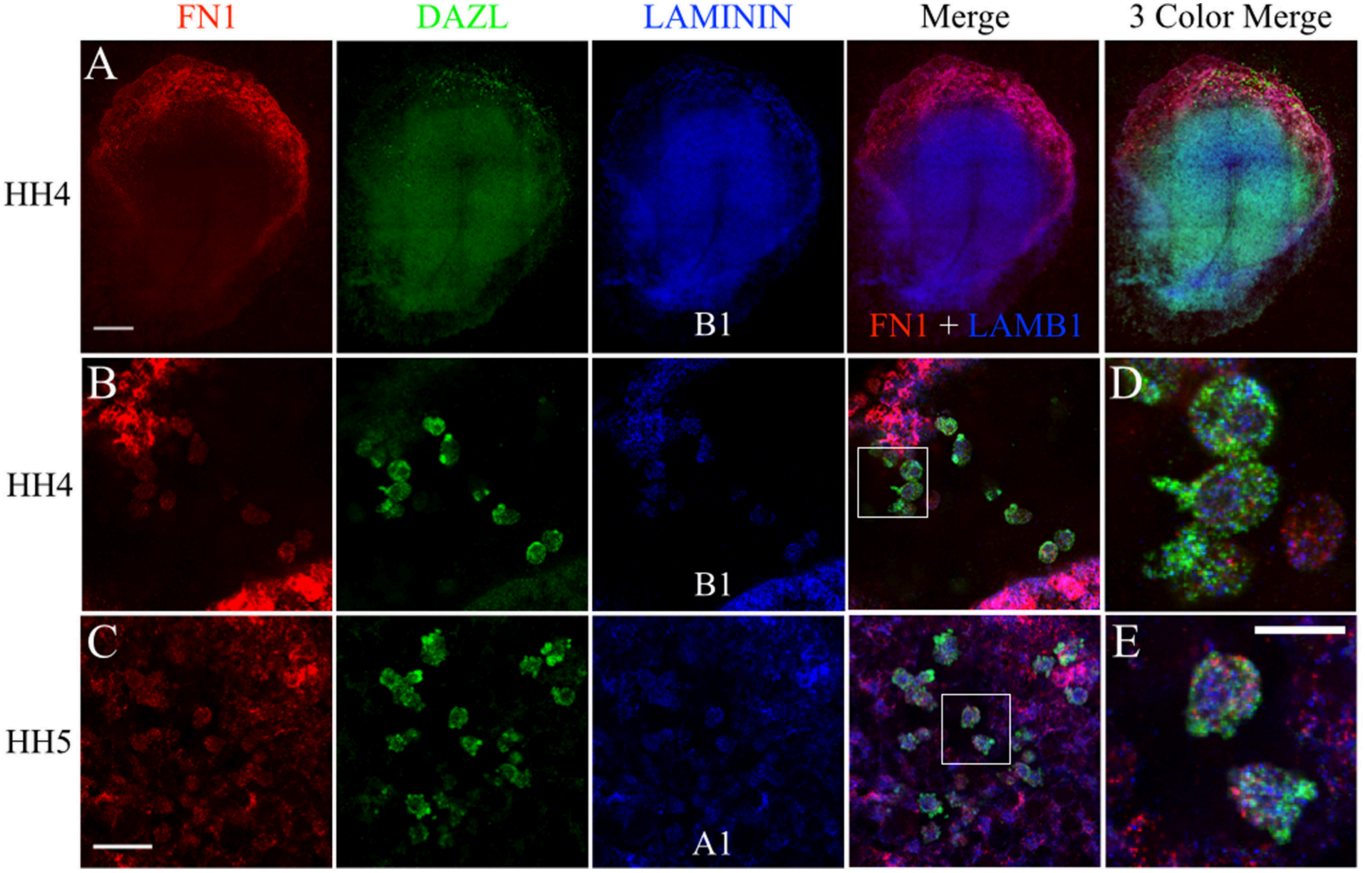
PGCs and their surrounding cells in the germinal crescent co-express transcripts for fibronectin and 2 subunits of laminin. mRNA transcripts for FN1, DAZL, LAMA1, and LAMB1 are expressed by PGCs and their surrounding cells within the germinal crescent of HH4 quail embryos. Wholemount multiplex hybridization chain reaction (HCR) for fibronectin1 (FN1), laminin alpha1 (LAMA1), laminin beta1 (LAMB1), and DAZL mRNA transcripts in HH4-5 quail embryos. **(A)** HH4. Tiled 10x confocal Z stack maximum intensity projections. From left to right: FN1 (red), DAZL (PGC marker, green), LAMB1 (blue), FN1 and LAMB1 Merge, FN1, DAZL, LAMB1 Merge. Scale bar = 500 μm. **(B,C)** Images are single optical sections (3.2 um each) from 20x confocal Z stacks with 2x zoom of HH4 **(B)** and HH5 **(C)** quail germinal crescent. From left to right: FN1 (red) DAZL (green) LAMB1 or LAMA1 (blue), 3 Color Merge of FN1, DAZL, and LAMB1. Scale bar = 25 μm. **(D)** Bounding box from B merge, at higher magnification. **(E)** Bounding box from C merge, at higher magnification. Scale bar = 20 μm.

The fluorescent *in situ* hybridization chain reaction (HCR) technique allows for multiple target mRNA transcripts to be detected simultaneously in whole-mount embryos (Choi et al., 2014). The anti-sense probe set sequences for quail FN1, LAMA1, LAMB1, ITGB1, and DAZL can be found in Figure S3. FN1 mRNA was widely detected across the embryo but showed particularly high expression in the anterior embryonic/extra-embryonic border area (Figure 8A). DAZL labeling localized a large concentration of PGCs in this region as well. LAMB1 was also ubiquitously expressed but was most prominent in the embryonic tissues, likely due to its abundance in the basement membrane on the ventral surface of the epiblast layer (Figure 8A; Zagris et al., 2000). Stage HH4-5 germinal crescent PGCs, and the nearby somatic cells, showed co-localized punctate labeling of FN1 with LAMB1 and LAMA1 (Figures 8B–E).

**Figure 9:**
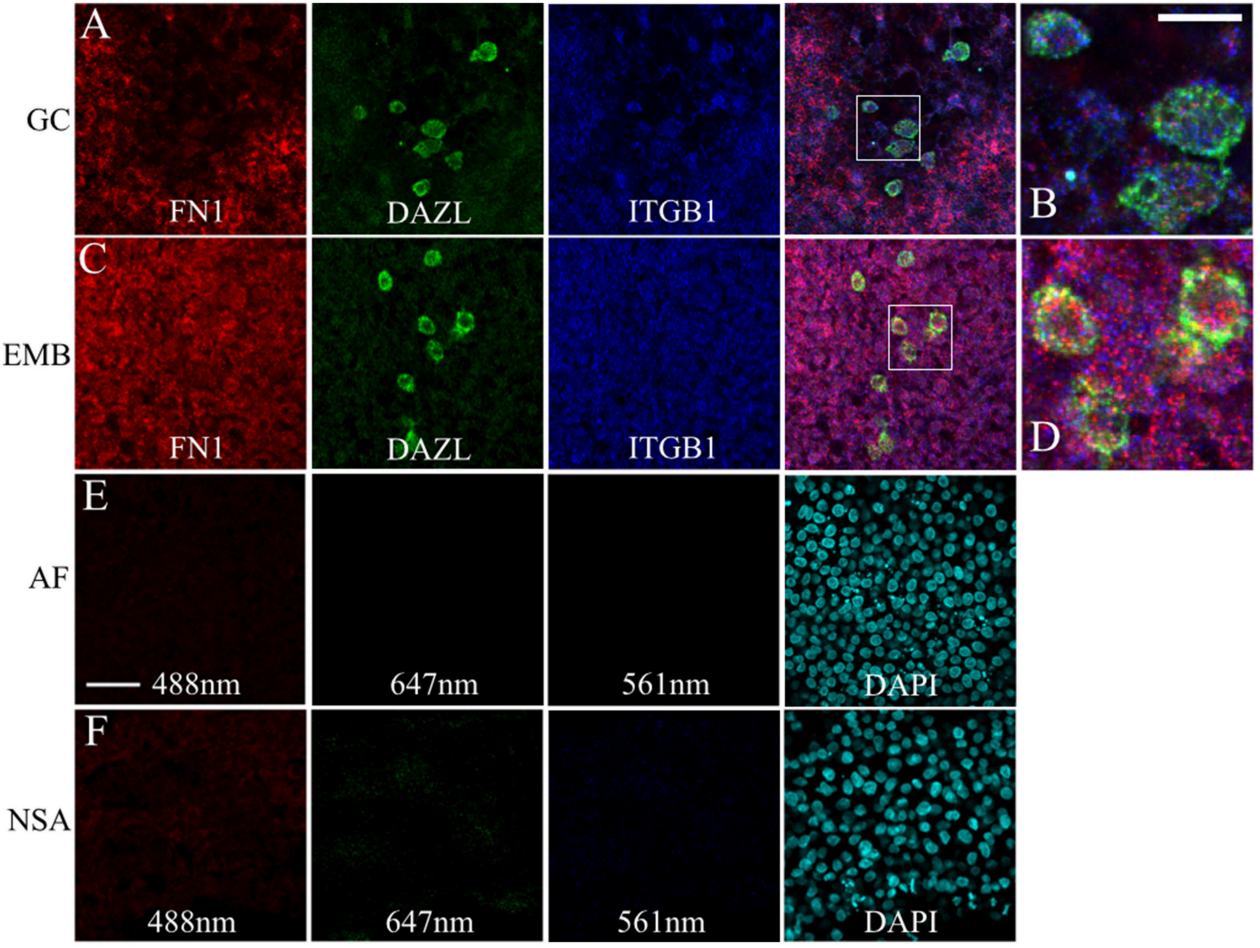
PGCs and their surrounding cells in the germinal crescent co-express transcripts for fibronectin and integrin beta 1. mRNA for fibronectin, DAZL and integrin beta1 receptor subunit are localized to PGCs and their surrounding cells in both the extra-embryonic germinal crescent and in the embryo proper. Whole-mount multiplex hybridization chain reaction (HCR) for Fibronectin1 (FN1, red), Integrin beta1 subunit (ITGB1, blue), and DAZL (green) mRNA transcripts in HH4 quail embryos. All images are single optical sections (3.2 μm each) from 20x confocal Z stacks with 2x zoom. **(A,B)** GC = germinal crescent. **(C,D)** EMB = Embryo. Mesoderm/hypoblast in the mid-lateral region of the embryo containing PGCs that have not yet reached the germinal crescent. **(B)** Bounding box from A at higher magnification. **(D)** Bounding box from **(C)** at higher magnification. 20 μm scale bar in **(B)** also applies to **(D)**. **(E)** AF = Auto-fluorescence control and **(F)** NSA = Non-specific amplification controls using the indicated laser lines. Control imaging conditions were identical to those used in **(A–D)**. Twenty five micrometer scale bar in **(E)** also applies to **(A,C,F)**.

Likewise, FN1 mRNA was co-expressed along with ITGB1 transcripts in germinal crescent PGCs (Figures 9A–D). Cells surrounding the PGCs also showed strong expression of fibronectin and integrin β1 mRNA transcripts. Control embryos incubated without probes and amplifying hairpins (Figure 9E) or hairpins alone (Figure 9F) failed to show the expression pattern of the experimental embryos.

### Time-Lapse Imaging of PGC-ECM Interactions

**Figure 10:**
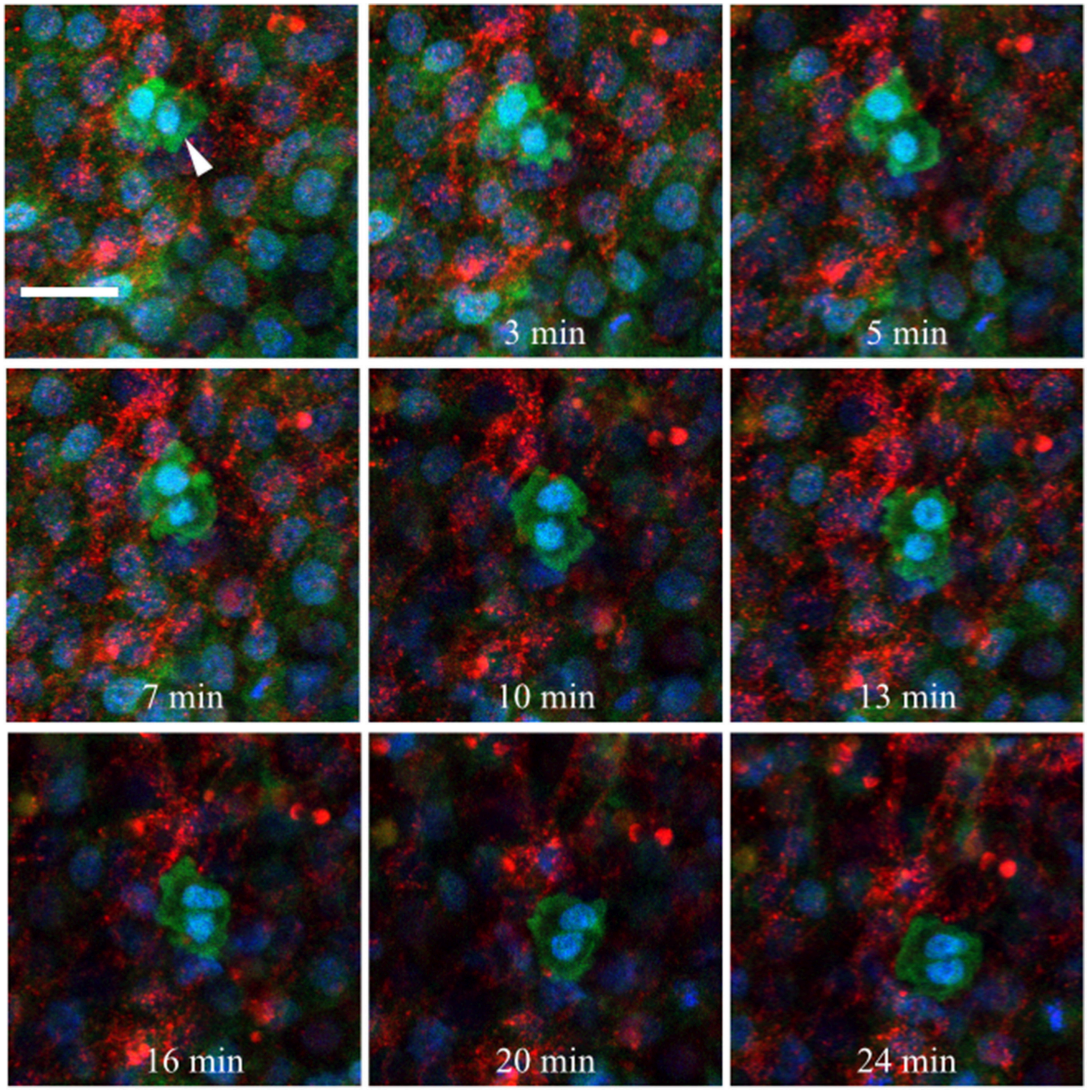
Germinal crescent PGCs are actively motile in the presence of fibronectin. Captured frames from live confocal microscope imaging of an HH5 [Tg(hUbC:H2B-Cerulean-2A-Dendra2)] quail embryo that was injected with an antibody against fibronectin (abB3/D6). This antibody was directly conjugated to AlexaFluor 594, red). The 20x images, with 2x optical zoom, are maximum intensity projections of 3 optical slices (2.3 μm each) taken from the dorsal aspect at 1.5 min intervals. Cell nuclei = H2B-Cerulean (blue), cell bodies = Dendra2 (green). While the cells are ubiquitously labeled by the transgene, those with low proliferation rates, such as PGCs (white arrow), are brighter than the surrounding cells. Scale bar = 25 μm. View the full movie in Video S1.

Fluorescently conjugated anti-glycoprotein antibodies were injected into the anterior AO/AP boundary of HH3 [Tg(hUbC:H2B-Cerulean-2A-Dendra2)] quail embryos in order to dynamically image PGC/ECM interactions in real time (Little and Drake, 2000). Figure 10 shows 9 captured time points (90 s intervals) from the germinal crescent of an HH5 transgenic embryo injected with anti-fibronectin (abB3/D6, AlexaFluor 594). The fibronectin matrix appears as bright red puncta and fibrils, cells are displayed with blue nuclei (H2B-Cerulean) and green cytoplasm (Dendra2 fluorescent protein). While the overall tissue and fibronectin fibrils move smoothly to the lower left, toward the embryo, the PGCs (large bright cells indicated with a white arrow, 0 min) show a rapid, constantly shifting cellular morphology (see Video S1). Blebs can be seen in most time points. Fibronectin fibrils surround the outside of the PGC cell closely, with the PGC cell body moving around and between fibrils.

**Figure 11:**
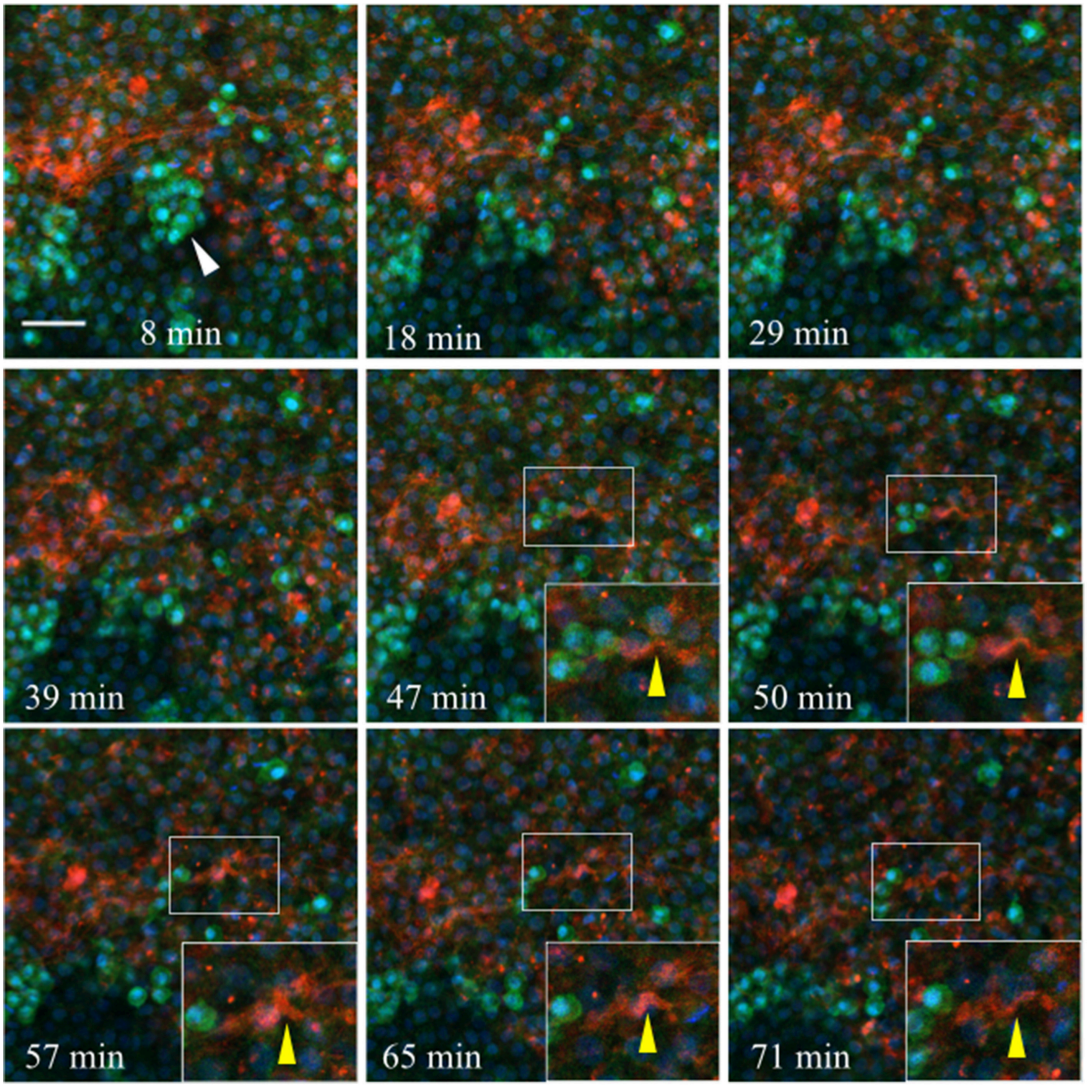
Germinal crescent PGCs are actively motile in the presence of laminin. Captured frames from live confocal microscope imaging of an HH4 [Tg(hUbC:H2B-Cerulean-2A-Dendra2)] quail embryo that was injected with an antibody against laminin (ab31-2). This antibody was directly conjugated to AlexaFluor 555, red). The 20x images are maximum intensity projections of 3 optical slices (3.2 μm each) taken from the dorsal aspect at 1.5 min intervals. Cell nuclei = H2B-Cerulean (blue), cell bodies = Dendra2 (green). PGCs (clump, white arrow), are brighter than the surrounding cells. Bounding box indicates the region shown in the lower right corner of the last five frames. The laminin fibril bundle (yellow arrow) has a smooth wave shape at 47 min. At 50–57 min the fibril bundle is condensed, pushing it upwards into a sharp peak. Later, as the tissue expands from 65 to 71 min, the bundle resumes its prior form. Scale bar = 25 μm. View the full movie in Video S2.

Injected anti-laminin antibodies (ab31-2, AlexaFluor 555) show a similar pattern when injected into the germinal crescent region of an HH4 transgenic embryo (Figure 11 and Video S2). The PGCs are mostly clustered and show the same fast morphology changes seen in Figure 10 and Video S1. The laminin ECM matrix can be seen shifting, expanding and contracting along with the large-scale tissue motion. The bounding boxes, starting at minute 47 (yellow arrow in Movie 2), highlight a small region of condensed laminin fibrils that begin in a relaxed, smooth wave-like configuration. The fibrils then seem to undergo a compression which forces the smooth wave into a sharp peak (minute 57). Next, the ECM and tissue seem to expand, bringing the laminin fibrils back into a smooth wave appearance by minute 71. All along, the PGCs move with the tissue flow but continue their independent, seemingly random shape changes.

Video S3 was taken from the lateral germinal crescent region of a single HH4 embryo injected with anti-fibronectin fluorescent antibodies. In this short example, the fibronectin fibrils can be seen as forming a loose band running roughly in the anterio-posterior direction (the embryo is toward the lower right, out of view). The PGCs, which are clustered to the medial side of the FN, move with the overall tissue/ECM flow laterally with only a few PGCs interacting closely with the ECM. A yellow arrow highlights a rapidly moving PGC that moves around the end of a FN fibril and comes to a stop along a second FN band. Video S4 begins a short time later at the same location of Video S5. Here, the impression is one of the FN forming a structural boundary, or demarcation line, across which the PGCs are not readily crossing, at least in the time frame imaged (about 60 min total). Video S5 shows a digitally zoomed view of a region from Video S4. The constant and sometimes erratic movements of individual PGCs can be seen, all occurring against the large-scale tissue and ECM flow. Lastly, Video S6 shows a dynamically moving meshwork of FN fibrils and puncta in the germinal crescent of an HH4 embryo demonstrating the fluid-like capacity of the ECM to shift, expand and contract with the large-scale tissue movements of the growing embryo.

### Perturbation of PGC Migration and ECM Expression by CSAT

**Figure 12:**
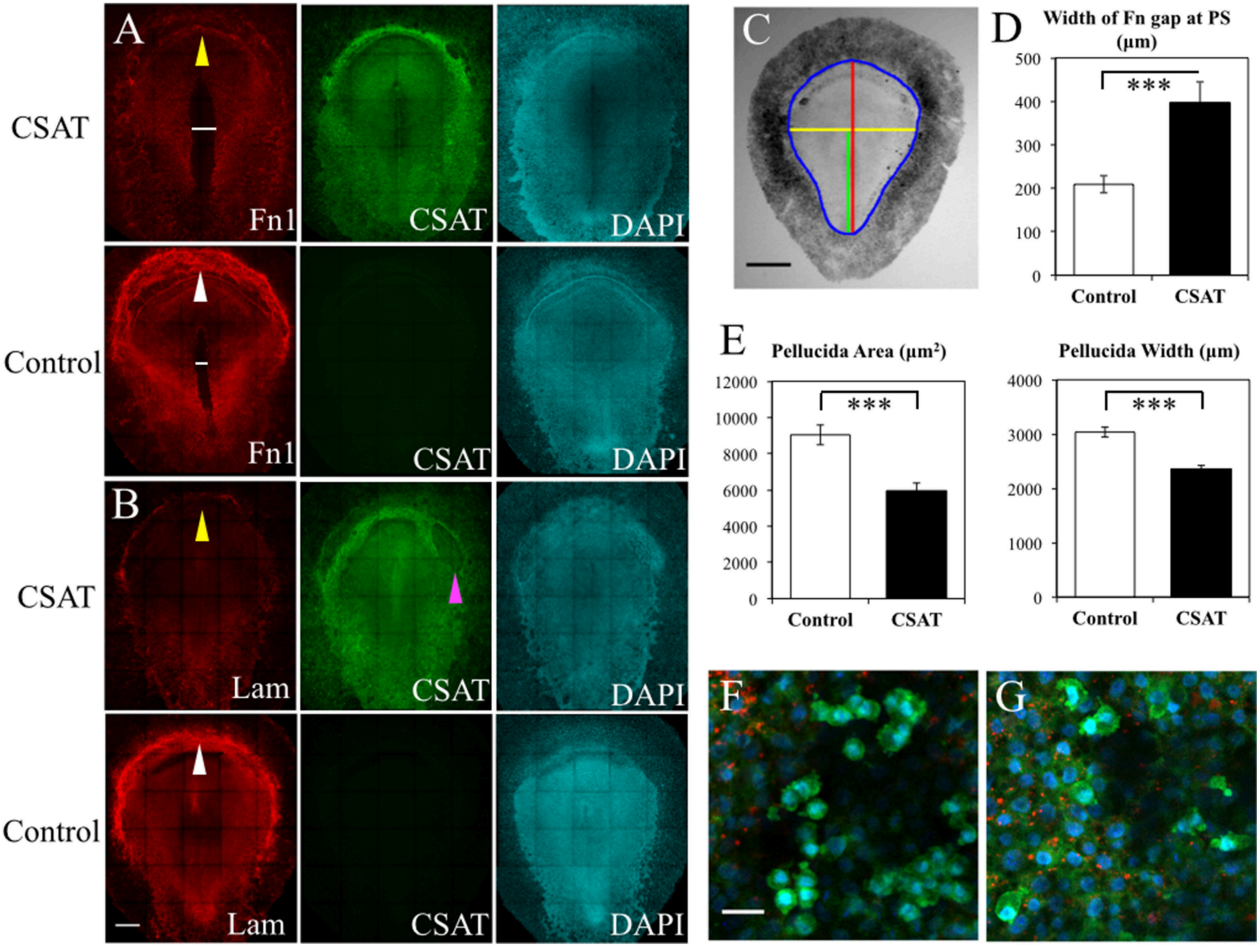
CSAT antibody decreases the amount of fibronectin in the germinal crescent. Whole-mount immunofluorescence for fibronectin (abB3/D6), and laminin (ab31-2) in HH5 quail embryos following injection of CSAT antibodies or control serum. All images are maximum intensity projections of tiled 10x confocal Z stacks taken from the dorsal aspect. **(A)** From left to right: fibronectin, CSAT antibody and DAPI cell nucleus marker of a CSAT injected (first row) and a serum injected control embryo (second row). The line in A indicates the region around the primitive streak that lacks fibronectin compared to the same region in the control embryo below. **(B)** Same pattern as **(A)** but with laminin immunofluorescence. White arrows in control fibronectin and laminin labeled embryos indicate the germinal crescent region with widespread mature ECM fibrils compared to the same regions in CSAT injected embryos (yellow arrows). The magenta arrow in **(B)** CSAT indicates a region where the ectoderm/epiblast has separated from the underlying mesoderm, a pattern which was seen in several CSAT injected embryos. Scale bar = 500 μm. **(C)** Bright-field stereomicroscope image of a control embryo showing the histomorphometry parameters measured. Blue = area pellucida and area opaca perimeter. Red = length of the area pellucida along the central axis. Yellow = width of the area pellucida at Hensen's node. Green = length of the primitive streak (PS) from Hensen's node to the caudal boundary of the area opaca. Scale bar = 1 mm. **(D–E)** Control vs. CSAT embryo group means ± standard error of the mean for histomorphometry measurements. **(D)** width between the fibronectin rich areas lateral to the PS in the tissue layers ventral to the epiblast. **(E)** Pellucida area (left) and pellucida width (right). Two tailed, paired Student's *T*-Test. ^***^P < 0.001. For additional measures, see Video S5. **(F)** Captured frame from time-lapse imaging of a HH5 [Tg(hUbC:H2B-Cerulean-2A-Dendra2)] before injection with CSAT antibodies. **(G)** Same embryo as in D, imaged post-CSAT antibody injection. Fibronectin (red), Dendra2 (green), H2B-Cerulean (blue). See Videos S7, S8 for the full time-lapse movies. PGC motility morphology appears unchanged after CSAT antibody injection. Scale bar = 25 μm.

To elucidate the functional roles that integrin receptors play in PGC movements, we introduced the CSAT (cell substrate attachment) antibody, which blocks the ability of integrin β1 containing receptors to bind their ECM components including laminin and fibronectin (Drake and Little, 1991; Drake et al., 1991). CSAT antibodies, or control medium, was injected under the epiblast of HH3 embryos, incubated for 6 h until HH5 and fixed. Detection of the CSAT antibody with fluorescently labeled secondary antibodies showed that the integrin β1 blocking immunoglobulins had spread across the entire embryonic and extra-embryonic area (Figures 12A,B). Control embryos, which lacked CSAT antibodies, showed no fluorescent signal. IF using anti-FN (abB3/D6) or laminin (ab31-2) antibodies revealed that CSAT injection had noticeably decreased the expression of both FN and laminin fibrils in the AO/AP boundary, particularly in the germinal crescent (Figures 12A,B, yellow arrows in CSAT embryos, white arrows in control embryos). In addition, the pattern of FN expression was altered in the CSAT injected embryos themselves. The fibronectin-free zone that typically surrounds the primitive streak was significantly wider in the CSAT treated embryos (Figures 12A,D, white line. *P* > 0.001, paired Student's *T*-Test). This result suggests that the mesodermal cells migrating laterally away from the primitive streak had not assembled mature FN fibrils as normal. Surprisingly, in both control and CSAT embryos, PGCs similarly moved from the embryonic regions to the extra-embryonic germinal crescent as in normal development (data not shown).

To rule out the possibility that these observed changes in ECM distribution were simply the result of overall delayed development caused by the CSAT antibody injection procedure, we made a series of measurements from bright-field stereomicroscope images of both the experimental and control embryos (Figure 12C). Both the overall area of the area pellucida (AP) and the width of the AP taken at Hensen's node were significantly smaller in the CSAT embryos (*P* > 0.001, paired Student's *T*-Test. Figure 12E). Overall AP length was also reduced in the CSAT embryos, as was primitive streak (PS) length (Figure S4). However, a careful staging of the embryos using the morphological landmarks described by Streit and Stern (2008) showed that all embryos, from both groups, had reached between HH4- and HH5- (Figures S4A,B). The ratio between AP length/PS length were also not different. Therefore, it appears that the CSAT treated embryos were not simply developmentally younger and hence, smaller. Adhesion between tissue layers was compromised, as evidenced by the frequent areas of tissue layer separation in CSAT treated embryos (magenta arrow, Figure 12B).

Interestingly, the overall distribution of PGCs, as detected by anti-DAZL antibodies, was not altered by CSAT when compared to serum-injected controls. To further test whether the CSAT antibodies could affect the cell motility and rapid cell morphology changes of PGCs, which were evident in our initial time-lapse images (Videos S4–S8), we conducted a second CSAT injection experiment. Five HH3 [UbC.CerD2] quail embryos were first injected with fluorescent anti-FN antibodies along the anterior AP/AO border and allowed to develop *in ovo* for 3 h. The embryos were then mounted on paper rings, cultured dorsal side down on a thin bed of agar-albumin and dynamically imaged to record the pre-CSAT PGC movements (Figure 12F and Video S7). CSAT antibodies were then injected into the mesodermal space at three locations along the germinal crescent. A small amount of fast green mixed with the serum allowed the visualization of the antibodies as they spread. The embryo was allowed to recover for 2 h before imaging again (Figure 12G and Video S8). A qualitative assessment of the PGC cell motility movements determined that the rapid shape changes and the appearances and disappearances of putative blebs, filopodia etc., had not been altered by the CSAT antibody (compare the movies in Videos S7, S8).

## Discussion

### Germinal Crescent as a Stem Cell Niche

We have shown that the complexity of the ECM fibril meshwork in the germinal crescent increases from HH3 to HH6, which corresponds with the arrival and concentration of PGCs in this anterior AP/AO border area (Figures 4–6; Critchley et al., 1979). Our results indicate that PGCs are actively contributing to the ECM in the germinal crescent. Whether the germinal crescent could be considered a stem cell niche is an open question. Certainly, the ECM has been shown to be important for determining the structural microenvironment of adult stem cell niches (reviewed in Watt and Huck, 2013). Our immunofluorescence and live imaging data suggest that this may be the case for the embryonic germinal crescent as well.

Unlike adult stem cell niches that are sometimes maintained for years, the embryonic germinal crescent is a highly transitory morphological space that is maintained from roughly HH3 to HH9, a time period of about 16 h. During this time, the vast majority of PGCs gather in this avascular region and closely interact with one another. By HH9 the vascular plexus, which has been concurrently forming by vasculogenesis in a posterior to anterior direction in both the left and right lateral extra-embryonic tissues, begins to converge toward the midline of the germinal crescent. By HH12, this process is complete and most PGCs have intravasated from the germinal crescent into the newly formed blood vessels. There is a multifaceted array of direct and indirect stem cell/ECM interactions that occur in an adult stem cell niche that regulate many processes including stem cell proliferation and differentiation (Rozario and DeSimone, 2010; Ahmed and Ffrench-Constant, 2016; Cant et al., 2016). It will be interesting to explore whether this bi-directional communication system is also present in the embryonic germinal crescent. In particular, whether or not the germinal crescent ECM is binding morphogens, chemokines or growth factors as a way of attracting the PGCs to the “correct” location, maintaining their pluripotency and contributing to the expansion of the vascular plexus is unknown. If so, it would lend additional support to the idea of Gu et al. (2009) that PGCs migrate along a “traveling niche” during embryogenesis.

### Flexibility of ECM/Integrin Interactions

The inability of the CSAT perturbation experiment to alter either the short-term cell motility or long-term migration of PGCs to the germinal crescent was surprising (Figure 12, and Videos S7, S8). Pertubation of integrin receptor binding to the ECM has led to reduced chicken neural crest cell migration (Bronner-Fraser, 1985). CSAT antibodies have also been shown to alter somite morphology and their lateral displacement from the neural tube in quail embryos (Drake and Little, 1991). In addition, CSAT antibodies reduced the embryo's normal rate of expansion, and the pattern of fibronectin expression around the primitive streak. Harrisson et al. (1993) found that microinjection of integrin receptor binding antibodies into early stage chicken embryos disrupted the ability of ingressing epiblast cells to spread laterally, resulting in an abnormal thickening of the primitive streak. Davidson et al. (2002) reported a similar lack of mesendoderm extension in *Xenopus laevis* explants treated with blocking antibodies to fibronectin or α5β1 integrin receptors. It is likely that the CSAT perturbation assay had a similar affect here and yet the PGCs continued their migration to the germinal crescent with no apparent delay.

By blocking the β1 integrin receptors' ability to bind either fibronectin or laminin we have likely demonstrated the inherent flexibility and redundancy of the integrin receptor/ECM system (Rozario and DeSimone, 2010). It is possible that other integrin receptor subunits may have compensated for the loss of β1 activity. Our PGC scRNAseq data show that after β1, the highest number of reads at HH3 and HH6 are subunits β7 and β8 (Figure 3B). At HH6, just after our CSAT experimental timeline, the expression levels of integrin β2–β6 subunits are low and highly variable from cell to cell, yet are at detectable levels. This expression appears transient, as these transcripts fail to appear in the other time points examined (Figure S5B). As for the integrin α subunits that can pair with non-β1 subunits, α4, and α6 show the highest expression levels with αV being detected at much lower levels (Figure S5A). Taken together, this would leave the possible compensating integrin receptors that bind fibronectin and/or laminin as α4β7, αVβ3, αVβ6, αVβ8, and α6β4. mRNA for α4β7 receptor subunits are consistently present in migrating avian PGCs at all ages studied (Figure 3). However, the α4β7 subunit has previously been described primarily in leukocytes and endothelial cells (Brezinschek et al., 1996), so its presence in our early time-point PGC transcriptome was un-expected and its potential role in PGC motility and migration is unknown. Likewise, the expression pattern and functionality of the other putative integrin receptors on migratory PGCs will have to be determined empirically.

Interestingly, integrin α5 subunit mRNA was not detected in the PGC transcriptome. This gene is expressed in early stage chicken embryos as shown in Muschler and Horwitz (1991) and Endo et al. (2013). Although chicken integrin α5 subunit was fully sequenced in 2013, the less complete nature of the *Coturnix* genome annotation has likely made integrin α5 subunit mRNA difficult to locate with automatic genomic algorithms (Endo et al., 2013). In this case, a directed BLAST search of the quail genome using the chicken sequence for ITGA5 failed to locate this gene. The *Coturnix* genome was recently sequenced and is currently being annotated based largely on the *Gallus* genome. It is likely that some genes found in chicken but not quail may simply be due to incomplete sequencing or lack of proper gene annotations in the nascent *Coturnix* genome. In other cases, the differences in genomes will reflect the true genetic diversity between these species. Discerning between these two possibilities will require additional investigation on a gene by gene basis.

Loss-of-function experiments can be difficult to interpret given the enormous complexity of the ECM/integrin receptor system, and other explanations for our CSAT perturbation results are equally plausible. It has been argued that PGCs initially utilize a passive mechanism of cell migration from EGK-X to HH2 and only when reaching the final anterior one-third of the embryo at HH3 do they employ active migration to reach the germinal crescent (Ginsburg and Eyal-Giladi, 1986; Kang et al., 2015). Perhaps by HH4-5, the stages examined in our perturbation assay, the PGCs were simply not actively utilizing integrin β1 receptors and fibronectin/laminin ECM fibrils as migratory and cell motility substrates. Injecting the CSAT at earlier stages may be a more effective strategy for blocking the ability of PGCs to populate the germinal crescent. Although it seems likely, whether the PGCs contributed to the reduction in laminin and fibronectin fibril meshwork in the germinal crescent after CSAT perturbation was not definitively shown in our current experiments. Performing scRNAseq after perturbation would help elucidate whether the transcriptome of the PGCs was altered, thus contributing to the re-modeling of the ECM in the germinal crescent.

Based on the Kyoto Encyclopedia of Genes and Genomes (KEGG) Pathway ECM-Receptor Interaction 04512 (www.kegg.jp) it is clear that non-integrin cell surface receptors in the proteoglycan, glycoprotein and immunoglobulin superfamily classes may play a significant role in PGC motility and migration. Transcript levels for non-integrin receptor genes with at least moderate levels of expression in the majority of PGCs are shown in Figure S5C. Syndecan2, DAG1 (dystroglycan associated glycoprotein 1), HMMR (hyaluronan mediated motility receptor) and CD47 all show significant transcript levels in PGCs. In addition, matrix proteins besides fibronectin and laminin such as agrin (AGRN, Figure 2A) may be involved in PGC motility and migration (Wei and Liu, 2014). Mean transcript numbers for additional matrix genes that showed at least moderate levels of expression in most of the sampled PGCs are reported in Figure S5D. While scRNAseq gives us a valuable snapshot of which mRNA transcripts are present in a cell at any given time, no insight into RNA processing, translation, post-translational modification or the level of functional protein is gained. Determining precisely which of these receptors and matrix proteins play an active role in PGC motility and migration will require additional experimentation. For instance, it has been shown that the syndecan family of membrane-intercalated proteoglycan receptors cooperate closely with integrin receptors and the ECM through multiple molecular signaling pathways to control cell adhesion, angiogenesis, axon guidance, wound healing, and cell migration (Morgan et al., 2007). The presence of Syndecan2 and multiple integrin receptor heterodimer subunits in our scRNAseq data suggests that integrin-syndecan functional synergy may play an important role in PGC directional migration as well.

The clear reduction of mature fibronectin and laminin fibrils in the germinal crescent of CSAT injected embryos highlights the role of integrin β1 subunit receptors in fibrillogenesis and matrix assembly (Figure 12). Loss-of-function experiments in cell lines have established the role that integrin β1 and β3 play in fibronectin matrix assembly (Darribère et al., 1990; Danen et al., 2002). We have shown that blocking integrin β1 subunit receptors reduces the fibronectin fibril meshwork pattern on a tissue-wide scale as well. How the β1 integrin receptors affect laminin assembly in the germinal crescent may be an interesting line of investigation given the presence of DAG1 and HSPG2 (heparan sulfate proteoglycan 2 or perlecan) in germinal crescent PGCs (Figures S5CD). Dystroglycan and perlecan are known to modify the assembly of laminin basement membrane in association with integrin β1 receptors (Henry et al., 2001; Soulintzi and Zagris, 2007; Nakaya et al., 2011). Whether these molecular interactions are contributing to assembly and modification of the laminin fibril meshwork in the germinal crescent during early development remains to be proven. Overall, the CSAT perturbation experiment has clearly highlighted the ability of cells and tissues to actively remodel their surrounding ECM using a diverse set of matrix molecules and receptors.

### ECM as an Autocrine/Paracrine System

Cells secret soluble fibronectin dimers into their extracellular environment. Fibronectin is then assembled into fibrils or basement membrane after binding to integrin receptors. These two processes can occur in the same cells (autocrine) or in different cells (paracrine), and are regulated in a spatio-temporal manner during development. It has been suggested by de Almeida et al. (2016) that fibronectin matrix assembly during early chick embryogenesis may function as either an autocrine or paracrine system depending on the germ layer and stage of development examined. This idea is based on growth factor signaling in which one tissue secretes the soluble factor and another tissue, the growth factor target tissue which may be located some distance away, expresses the receptor for it. In order to explore this question for migratory PGCs, we examined fibronectin protein and mRNA expression in PGCs taken from the first 2 days of development. scRNAseq shows that PGCs of all three stages (HH3, HH6, and HH12) are producing mRNA transcripts for both fibronectin and various integrin receptor subunits (Figures 2, 3). *In situ* hybridization by HCR imaging confirms that PGCs have similar expression patterns of fibronectin and integrin receptor mRNA transcripts as their neighboring somatic cells (Figures 8, 9). PGCs that are migrating within the mesodermal space of the embryo, and PGCs that have previously reached the extra-embryonic germinal crescent show similar expression patterns (Figures 9A–D). Likewise, both PGCs and their neighboring cells express integrin β1 receptor protein as shown in our IF data (Figure 7). Thus, consistent with an autocrine system of matrix regulation, PGCs not only produce soluble fibronectin but they also express the integrin receptors needed to initiate fibril assembly. It is likely that PGCs interact in a bi-directional way with their surrounding cells in order to secrete, assemble and re-model the fibronectin matrix dynamically during all phases of their migratory journey.

## Data Availability

All datasets generated for this study are included in the manuscript and/or the Supplementary Files.

## Ethics Statement

This study was carried out in accordance with the recommendations in the Guide for the Care and Use of Laboratory Animals of the National Institutes of Health. The protocol was approved by the University of Southern California (Protocol # 11944-CR001) and the Children's Hospital Los Angeles (Protocol # 351-16) Institutional Animal Care and Use Committees.

## Author Contributions

RL and JE planned the experiments. RL and SS prepared the quail matrisome. DH, MD, SS, SH, JW, and JS performed the experiments. RL, JK, JS, DH, and JE analyzed the scRNAseq dataset. DH and RL wrote the manuscript.

### Conflict of Interest Statement

The authors declare that the research was conducted in the absence of any commercial or financial relationships that could be construed as a potential conflict of interest.

## Supplementary Material

Figure S1 Freshly harvested [Tg(PGK1:H2B-mCherry)] quail embryos at the 3 developmental stages used for single cell mRNA isolation. Bright-field (BF, left) and red epi-fluorescence (right) stereomicroscope images. Dashed bounding boxes represent the approximate area of the embryo excised for tissue dissociation followed by single cell picking, mRNA amplification and sequencing. PS, primitive streak; HF, head fold. Rostral (anterior) to the top in all images. Scale bars = 1 mm.

Figure S2 Whole-mount immunofluorescence for fibronectin (B3/D6) and CVH in the germinal crescent and head region of an HH12 [Tg(PGK1:H2B-mCherry)] quail embryo. **(A)** Fibronectin. **(B)** CVH (PGC marker). **(C)** Fibronectin and CVH merged and **(D)** DAPI. Images are maximum intensity projections of 5x tiled confocal Z stacks taken from the dorsal aspect. Anterior (rostral) to top. **(A–D)** Scale bar = 500 μm. **(E–H)** taken at location of bounding box in **(B)**. **(E)** Fibronectin. **(F)** CVH. **(G)** H2B-mCherry. **(H)** Fibronectin, H2B-mCherry and CVH merged. Images are maximum intensity projections of 40x confocal Z stacks taken from the dorsal aspect. Anterior (rostral) to top. **(E–H)** Scale bar = 50 μm.

Figure S3 Anti-sense oligonucleotide sequences used for hybridization chain reaction (HCR) probes. This *in-situ* hybridization technique uses a unique initiator sequence for each probe set to allow for simultaneous multiplex hybridizations.

Figure S4 Measurements of quail embryos following injection of CSAT antibodies or control serum. **(A)** Embryos were staged based on the Hamilton-Hamburger chicken developmental series for both CSAT antibody injected (*n* = 11) and control embryos (*n* = 7). **(B)** Numerical values were assigned to the stages as follows: HH4– = 3.667, HH4 = 4, HH4+ = 4.333, HH5– = 4.667, HH5 = 5. Control vs. CSAT injected embryo group means ± standard error of the mean for histomorphometry measurements of area pellucida length/width ratio, primitive streak (PS) length and pellucida length/PS length ratio. Two tailed, paired Student's *T*-Test. ^*^*P* < 0.05. NS = not significantly different.

Figure S5 Transcriptome of less abundant ECM mRNA transcripts in PGCs across 3 developmental time points based on the KEGG Pathway ECM-Receptor Interaction 04512 for Japanese quail. **(A)** Integrin alpha subunit receptors. **(B)** Integrin beta subunits. **(C)** Non-integrin receptors. SDC, Syndecan (proteoglycan); DAG, Dystroglycan (glycoprotein); HMMR, Hyaluronan Mediated Motility Receptor and CD47 (immunoglobulin superfamily genes). **(D)** Matrix genes. HSPG, Heparan Sulfate Proteoglycan; FBLN, Fibrillin; FBN3, Fibulin. Bars represent the mean + s.e.m. The number of single cells in each group are shown in the legend.

Table S1 Japanese quail matrisome.

Table S2 Quail PGC transcriptome.

Video S1 Live confocal microscope imaging of the germinal crescent region of an HH5 [Tg(hUbC:H2B-Cerulean-2A-Dendra2)] quail embryo that was injected with an antibody against fibronectin (abB3/D6). The antibody was directly conjugated to AlexaFluor 594, red). The 20x images, with 2x optical zoom, are maximum intensity projections of 3 optical slices (2.3 μm each) taken from the dorsal aspect at 1.5 min intervals. Total elapsed time is 26 min. Cell nuclei = H2B-Cerulean (blue), cell bodies = Dendra2 (green). While the cells are ubiquitously labeled by the transgene, those with low proliferation rates, such as PGCs, are brighter than the surrounding tissue. Anterior to top. Embryonic tissue to the lower right. Extra-embryonic tissue to the upper left. Scale bar = 25 μm.

Video S2 Live confocal microscope imaging of the central germinal crescent region of an HH4 [Tg(hUbC:H2B-Cerulean-2A-Dendra2)] quail embryo that was injected with an antibody against laminin (ab31-2). This antibody was directly conjugated to AlexaFluor 555 (red). The 20x images are maximum intensity projections of 3 optical slices (3.2 μm each) taken from the dorsal aspect at 1.5 min intervals. Total elapsed time is 71 min. Cell nuclei = H2B-Cerulean (blue), cell bodies = Dendra2 (green). The PGCs are typically larger and brighter than the surrounding cells. Scale bar = 50 μm.

Video S3 Live confocal microscope imaging of the lateral germinal crescent region of an HH4 [Tg(hUbC:H2B-Cerulean-2A-Dendra2)] quail embryo that was injected with an antibody against fibronectin (abB3/D6). The antibody was directly conjugated to AlexaFluor 594, red). The 20x images are maximum intensity projections of 3 optical slices (2.3 um each) taken from the dorsal aspect at 1.5 min intervals. Total elapsed time is 18 min. Cell nuclei = H2B-Cerulean (blue), cell bodies = Dendra2 (green). PGCs are larger and brighter than the surrounding tissue and appear in clusters and individually. Anterior to top, mediolateral midline to the right out of view. Embryonic tissue to the lower right. Extra-embryonic tissue to the left and upper left. Yellow arrow denotes a PGC that moves rapidly around a FN fibril. Scale bar = 50 um.

Video S4 Live confocal microscope imaging of the lateral germinal crescent region of an HH4 [Tg(hUbC:H2B-Cerulean-2A-Dendra2)] quail embryo that was injected with an antibody against fibronectin (abB3/D6). The antibody was directly conjugated to AlexaFluor 594 (red). The 20x images are maximum intensity projections of 3 optical slices (2.3 μm each) taken from the dorsal aspect at 1.5 min intervals. Total elapsed time is 43 min. Cell nuclei = H2B-Cerulean (blue), cell bodies = Dendra2 (green). PGCs are larger and brighter than the surrounding tissue and appear in clusters and individually. Anterior to top, mediolateral midline to the right out of view. Embryonic tissue to the lower right. Extra-embryonic tissue to the left and upper left. Scale bar = 50 μm.

Video S5 Cropped area from the lower left region of the movie in Video S2 shown with increased magnification. Live confocal microscope imaging of the lateral germinal crescent region of an HH4 [Tg(hUbC:H2B-Cerulean-2A-Dendra2)] quail embryo that was injected with an antibody against fibronectin (abB3/D6). The antibody was directly conjugated to AlexaFluor 594 (red). The 20x images are maximum intensity projections of 3 optical slices (2.3 μm each) taken from the dorsal aspect at 1.5 min intervals. Total elapsed time is 43 min. Cell nuclei = H2B-Cerulean (blue), cell bodies = Dendra2 (green). PGCs are larger and brighter than the surrounding tissue and appear in clusters and individually. Anterior to top, mediolateral midline to the right out of view. Embryonic tissue to the lower right. Extra-embryonic tissue to the left and upper left. Scale bar = 25 μm.

Video S6 Live confocal microscope imaging of the central germinal crescent region of an HH4 [Tg(hUbC:H2B-Cerulean-2A-Dendra2)] quail embryo that was injected with an antibody against fibronectin (abB3/D6). The antibody was directly conjugated to AlexaFluor 594 (red). The 20x images are maximum intensity projections of 3 optical slices (2.3 μm each) taken from the dorsal aspect at 1.5 min intervals. Total elapsed time is 46 min. Cell nuclei = H2B-Cerulean (blue), cell bodies = Dendra2 (green). PGCs are larger and brighter than the surrounding tissue and appear in clusters and individually. Anterior to the top. Embryonic tissue toward the bottom. Extra-embryonic tissue at the top. Scale bar = 50 μm.

Video S7 Pre-CSAT antibody injection movie for embryo 2. Time-lapse confocal microscope imaging of the central germinal crescent region of an HH5 [Tg(hUbC:H2B-Cerulean-2A-Dendra2)] quail embryo that was injected with an antibody against fibronectin (abB3/D6). The antibody was directly conjugated to AlexaFluor 594 (red). After incubation, the germinal crescent region was live imaged to establish the baseline movement of the PGCs. The 20x images, with 1.5x optical zoom are maximum intensity projections of 3 optical slices (2.3 μm each) taken from the dorsal aspect at 1.5 min intervals. Total elapsed time is 28 min. Cell nuclei = H2B-Cerulean (blue), cell bodies = Dendra2 (green). PGCs are larger and brighter than the surrounding tissue and appear individually and in clumps in this movie. Anterior to the top. Embryonic tissue toward the bottom. Extra-embryonic tissue at the top. Scale bar = 50 μm.

Video S8 Post-CSAT antibody injection movie for embryo 2. See Video S7 for pre-CSAT injection cell movements. CSAT antibodies against integrin β1 subunits fail to affect the cell motility and morphology of PGCs in HH5 embryos. Time-lapse confocal microscope imaging of the central germinal crescent region of an HH5 [Tg(hUbC:H2B-Cerulean-2A-Dendra2)] quail embryo that was injected with an antibody against fibronectin (abB3/D6). The antibody was directly conjugated to AlexaFluor 594 (red). After incubation, the germinal crescent region was injected again, this time with CSAT antibodies (174 ug/ml) and imaged live. The 20x images, with 2x optical zoom are maximum intensity projections of 3 optical slices (2.3 μm each) taken from the dorsal aspect at 1.5 min intervals. Total elapsed time is 24 min. Cell nuclei = H2B-Cerulean (blue), cell bodies = Dendra2 (green). PGCs are larger and brighter than the surrounding tissue and appear individually and in clumps in this movie. Anterior to the top. Embryonic tissue toward the bottom. Extra-embryonic tissue at the top. Scale bar = 25 μm.

## Abbreviations

AF: auto-fluorescence
AO: area opaca
AP: area pellucida
CSAT: cell substrate attachment antibody
ECM: extra-cellular matrix
EGK: Eyal-Giladi and Kochav stage
EMB: embryo
FISH: fluorescence *in situ* hybridization
FN: fibronectin
FP: fluorescent protein
GC: germinal crescent
H2B: histone 2B
HH Hamburger Hamilton stage
HCR: hybridization chain reaction
hUbC: human ubiquitin C promoter
IF: immunofluorescence
ITG: integrin receptor
LAM: laminin
NA: numerical aperture
NSA: non-specific amplification
PGC: primordial germ cell
PGK: phosphoglycerate kinase promoter
scRNAseq: single cell RNA sequencing
Tg: transgene.
