# Supplementary figures and images for: Avian Primordial Germ Cells Contribute to and Interact With the Extracellular Matrix During Early Migration
Source: Front Cell Dev Biol. 2019 Mar 28;7:35. doi: 10.3389/fcell.2019.00035 (PMC6447691; doi:10.3389/fcell.2019.00035)

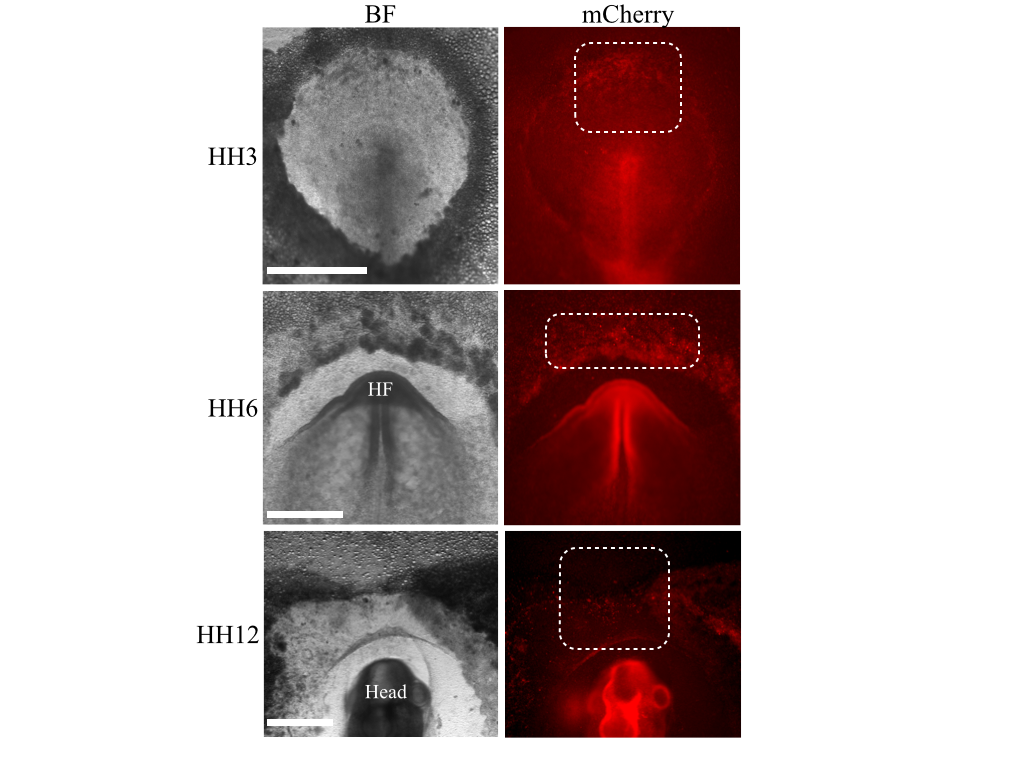

Supplement: Figure S1 — Freshly harvested [Tg(PGK1:H2B-mCherry)] quail embryos at the 3 developmental stages used for single cell mRNA isolation. Bright-field (BF, left) and red epi-fluorescence (right) stereomicroscope images. Dashed bounding boxes represent the approximate area of the embryo excised for tissue dissociation followed by single cell picking, mRNA amplification and sequencing. PS, primitive streak; HF, head fold. Rostral (anterior) to the top in all images. Scale bars = 1 mm. [file Image_1.TIFF]

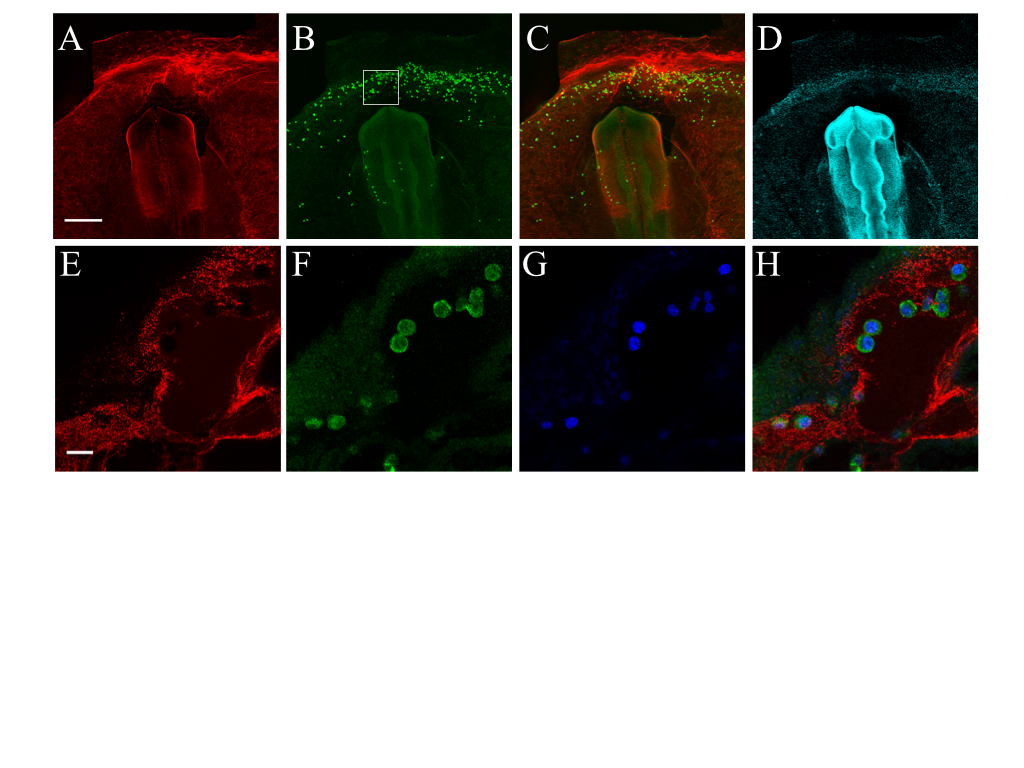

Supplement: Figure S2 — Whole-mount immunofluorescence for fibronectin (B3/D6) and CVH in the germinal crescent and head region of an HH12 [Tg(PGK1:H2B-mCherry)] quail embryo. (A) Fibronectin. (B) CVH (PGC marker). (C) Fibronectin and CVH merged and (D) DAPI. Images are maximum intensity projections of 5x tiled confocal Z stacks taken from the dorsal aspect. Anterior (rostral) to top. (A–D) Scale bar = 500 μm. (E–H) taken at location of bounding box in (B). (E) Fibronectin. (F) CVH. (G) H2B-mCherry. (H) Fibronectin, H2B-mCherry and CVH merged. Images are maximum intensity projections of 40x confocal Z stacks taken from the dorsal aspect. Anterior (rostral) to top. (E–H) Scale bar = 50 μm. [file Image_2.TIFF]

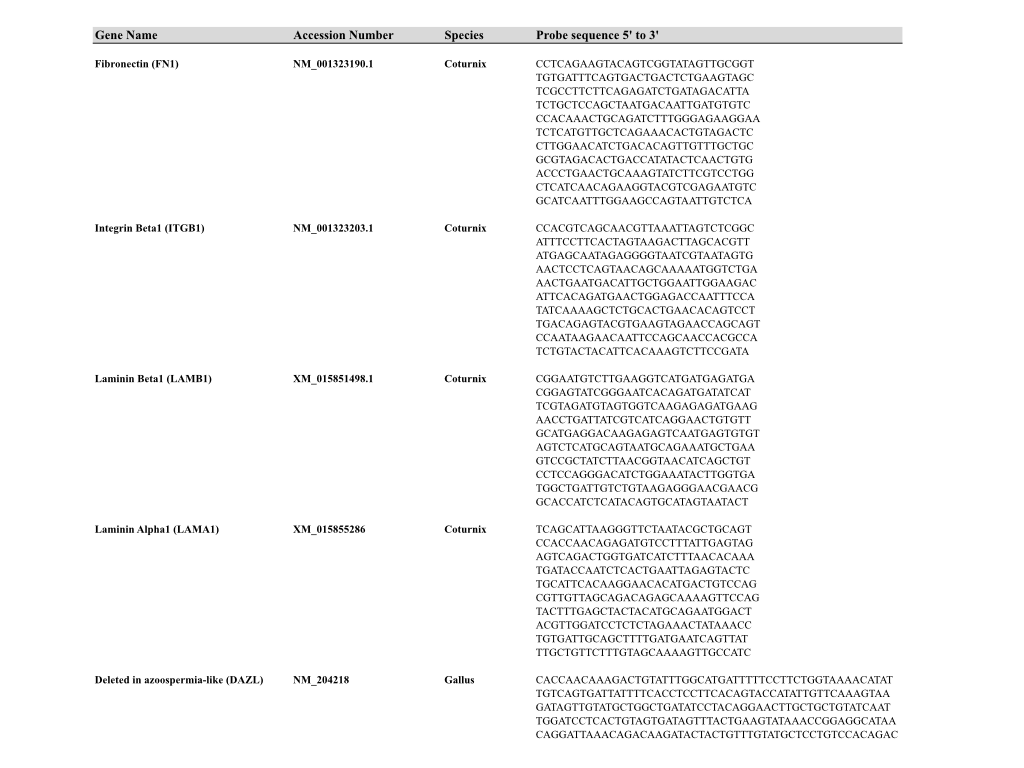

Supplement: Figure S3 — Anti-sense oligonucleotide sequences used for hybridization chain reaction (HCR) probes. This in-situ hybridization technique uses a unique initiator sequence for each probe set to allow for simultaneous multiplex hybridizations. [file Image_3.TIFF]

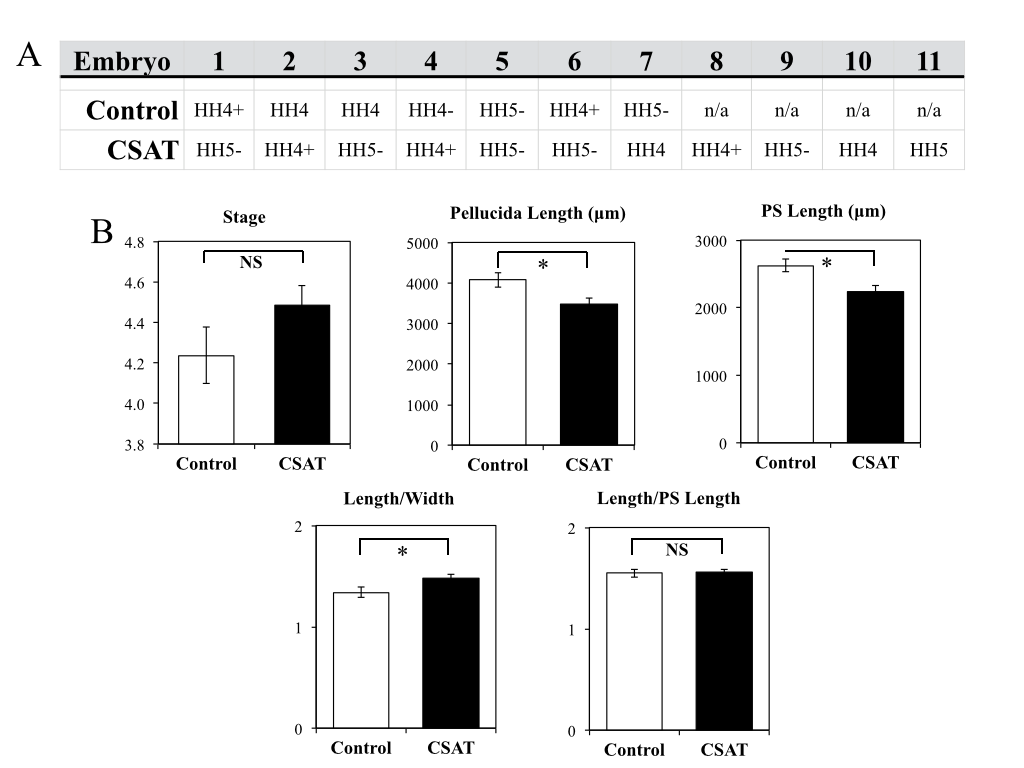

Supplement: Figure S4 — Measurements of quail embryos following injection of CSAT antibodies or control serum. (A) Embryos were staged based on the Hamilton-Hamburger chicken developmental series for both CSAT antibody injected (n = 11) and control embryos (n = 7). (B) Numerical values were assigned to the stages as follows: HH4– = 3.667, HH4 = 4, HH4+ = 4.333, HH5– = 4.667, HH5 = 5. Control vs. CSAT injected embryo group means ± standard error of the mean for histomorphometry measurements of area pellucida length/width ratio, primitive streak (PS) length and pellucida length/PS length ratio. Two tailed, paired Student's T-Test. *P < 0.05. NS = not significantly different. [file Image_4.TIFF]

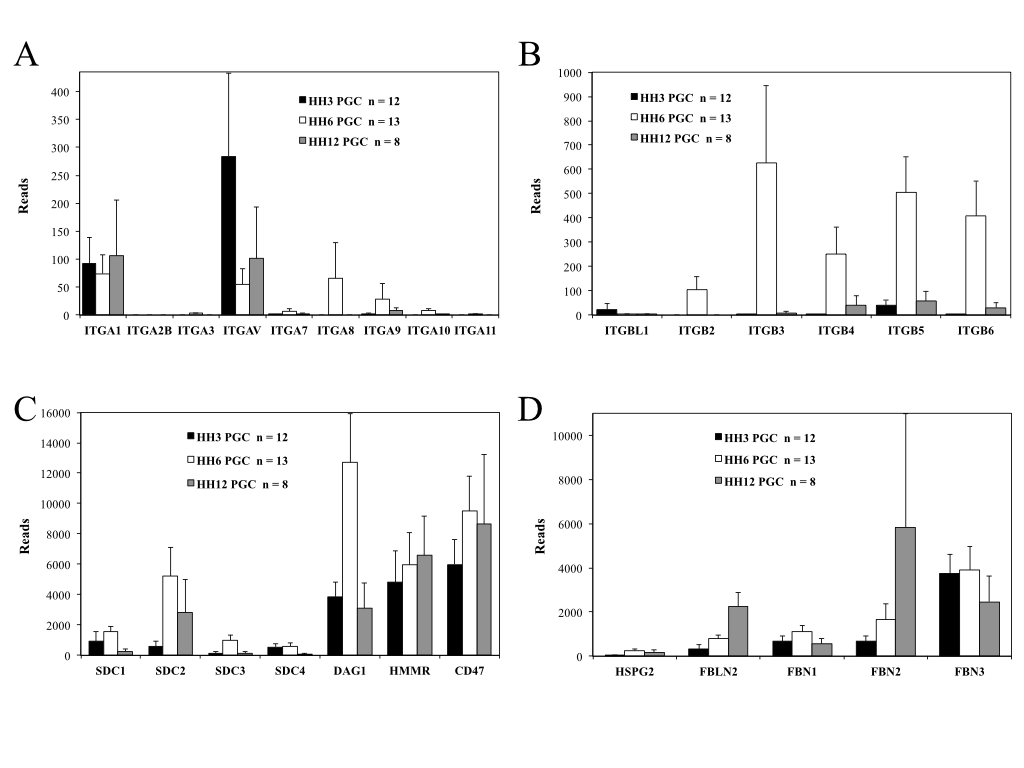

Supplement: Figure S5 — Transcriptome of less abundant ECM mRNA transcripts in PGCs across 3 developmental time points based on the KEGG Pathway ECM-Receptor Interaction 04512 for Japanese quail. (A) Integrin alpha subunit receptors. (B) Integrin beta subunits. (C) Non-integrin receptors. SDC, Syndecan (proteoglycan); DAG, Dystroglycan (glycoprotein); HMMR, Hyaluronan Mediated Motility Receptor and CD47 (immunoglobulin superfamily genes). (D) Matrix genes. HSPG, Heparan Sulfate Proteoglycan; FBLN, Fibrillin; FBN3, Fibulin. Bars represent the mean + s.e.m. The number of single cells in each group are shown in the legend. [file Image_5.TIFF]
